# Intracellular *Staphylococcus aureus* triggers isolate-specific host transcriptional responses alongside TNF-R1 regulated cell fate decisions

**DOI:** 10.1186/s12964-026-03246-8

**Published:** 2026-09-28

**Authors:** Annika Walter, Thorsten Bischler, Marvin Jungblut, Leonhard F. Breitsprecher, Filip Vilotic, Johanna Beck, Lucia Hofmann, Nicole Schäfer, Tanja Ziesmann, Rico Franzkoch, Silke Härteis, Iana Gadjalova, Ute Distler, Gerti Beliu, Olympia-Ekaterini Psathaki, Michael Hensel, Wulf Schneider-Brachert, Tom Gräfenhan, Thomas Stempfl, Bärbel Kieninger, Sabrina Mühlen, Volker Alt, Gopala-Krishna Mannala, Jürgen Fritsch

**Affiliations:** 1 Department of Infection Prevention and Infectious Diseases, University Hospital of Regensburg, University of Regensburg, Regensburg, 93053 Germany; 2 Core Unit Systems Medicine, University of Würzburg, Würzburg, Germany; 3 Rudolf-Virchow-Center for Integrative and Translational Bioimaging, University of Würzburg, Würzburg, Germany; 4 Center of Cellular Nanoanalytics Osnabrück, Integrated Bioimaging Facility, University of Osnabrück, Osnabrück, Germany; 5 Institute for Molecular and Cellular Anatomy, University of Regensburg, Regensburg, Germany; 6 Department of Orthopedic Surgery, Experimental Orthopedics, Center for Medical Biotechnology (ZMB), University of Regensburg, Regensburg, Germany; 7 Institute for Immunology, University Medical Center of the Johannes-Gutenberg University, Mainz, Germany; 8 Institute for Clinical Chemistry and Pathobiochemistry, School of Medicine, Technical University of Munich, Munich, Germany; 9 TranslaTUM, Center for Translational Cancer Research, Technical University Munich, Munich, Germany; 10 Regensburg Center for Ultrafast Nanoscopy, University of Regensburg, Regensburg, Germany; 11 Division of Microbiology, University of Osnabrück, Osnabrück, Germany; 12 Center of Excellence for Fluorescent Bioanalytics (KFB), University of Regensburg, Regensburg, Germany; 13 Department of Molecular Immunology, Ruhr-University Bochum, Bochum, Germany; 14 Department of Trauma Surgery, University Hospital Regensburg, Regensburg, Germany

**Keywords:** *Staphylococcusaureus*, Intracellular infection, Transcriptomics, TNF-α, TNF receptor 1, Host cell death, Apoptosis, Host response, Host‒pathogen interactions, Perinuclear

## Abstract

**Background:**

*Staphylococcus aureus* (*S. aureus*) is an increasingly recognized intracellular pathogen, yet infection outcomes vary with bacterial isolate and host cell type. Many mechanisms underlying these differences remain poorly understood. This study investigated how distinct intracellular *S. aureus* isolates influence host signaling programs and infection outcomes by modulating cell death pathways and TNF-R1 dependent regulation of host cell fates across different human cell lines.

**Methods:**

The intracellular localization of four *S. aureus* isolates was analyzed via transmission electron microscopy (TEM), structured illumination microscopy (SIM), serial block-face scanning electron microscopy (SBF-SEM), and imaging flow cytometry. The transcriptional reprogramming of infected U937 monocytes was examined via mRNA sequencing. Infection outcomes and early TNF-R1 surface expression were characterized and compared with those of A549 and SaOS-2 cell lines employing flow cytometry and Western blot analysis, characterizing host cell death mechanisms in both wild-type and TNF-R1‒deficient backgrounds.

**Results:**

All *S. aureus* isolates localized to the endo-lysosomal and cytosolic compartments but were also frequently detected in nucleo-proximal regions. In U937 monocytes, infection induced a conserved stress signature alongside isolate‑specific transcriptional programs divergently affecting inflammation, metabolism, and cell fate, which was markedly attenuated in response to the chronic‑infection isolate EDCC 5464. Similarly, cell death outcomes were isolate‑ and cell type-dependent, involved caspase activation consistent with intrinsic and extrinsic apoptosis signaling, and caspase-1 activation at distinct temporal dynamics. TNF‑R1 loss initially delayed but later increased later isolate-independent cytotoxicity. Higher TNF-R1 surface expression was triggered in U937 monocytes by all viable isolates, but only by EDCC 5464 in SaOS-2 and A549 cells. Viability of the latter was only marginally influenced by isolate or TNF-R1 loss.

**Conclusions:**

These results highlight the multilayered determinants governing intracellular *S. aureus* survival, host cell susceptibility, and noncanonical intracellular localization that warrants further investigation. The TNF/TNF-R1 axis appears to critically influence regulated host defenses during early infection stages in a tissue-specific manner, though its mechanistic contribution remains to be fully elucidated. Together with distinct isolate-driven gene expression profiles of U937 monocytes, infection risks under TNF-targeted therapies and the contribution of *S. aureus* heterogeneity should be considered in the design of future host-directed treatment strategies.

**Graphical Abstract:**

Isolate- and cell type- specific host responses to internalized *S. aureus* across subcellular localization, transcriptional programs, host fates, and the role of TNF-R1 signaling in infection outcomes of U937 monocytes. In U937_wt_, SaOS-2_wt_, and A549_wt_ cells, intracellular *S. aureus* resides in (frequently LAMP-1–decorated) membrane-enclosed compartments or directly in the cytosol at 2 hpi, but also close to host nuclear structures across cell lines. TNF-R1 surface expression is increased by all viable isolates in U937_wt_ cells, but only by EDCC 5464 in A549 _wt_ and SaOS-2 _wt_ cells. At 4 hpi, *S. aureus* triggers differential gene expression in (A) U937_wt_ cells to an isolate-specific extent, with both unique and shared transcriptomic signatures across the four isolates. This response is weakest for the chronic infection isolate EDCC 5464. Isolate-dependent cell death (marked by Annexin V/7-AAD staining; not shown) of U937_wt_ cells involves heterogenous activation of caspase-8, caspase-9 (EDCC 5055 only), and variable effector caspase-3/-7 activity in earlier stages of infection (6 hpi). The extent of activation then barely further increases until 24 hpi and includes changes in the mitochondrial trans-membrane potential (ΔΨm) and additional caspase-1 activation, consistent with engagement of apoptotic signaling alongside other pathways at distinct stages of infection. *S. aureus*-induced cell death and caspase activation is abolished in (B) U937_ΔTNF-R1_ at 6 hpi, but is significantly reinforced across the tested effectors differences at 24 hpi, whereas isolate-specific differences observed in U937_wt_ are lost. (C) SaOS-2 _wt_ cells show moderate caspase-3/-7 and -1 activation, while infection induces detachment of (D) A549_wt_ cells that correlates with Annexin V/-7-AAD staining (not shown) but is accompanied by minimal caspase activation. The number of coloured arrows indicate the mean proportion of effector-positive cells (↑ ~20-40%, ↑ ↑ 40-60%, ↑ ↑ ↑ >60%), the number of coloured plus-signs (+) indicate a trend for the extent of the effect; colours represent each *S. aureus *isolate: SH1000 (green), EDCC 5055 (blue), EDCC 5458 (orange), EDCC 5464 (yellow). Created with BioRender.com.

**Figure:**
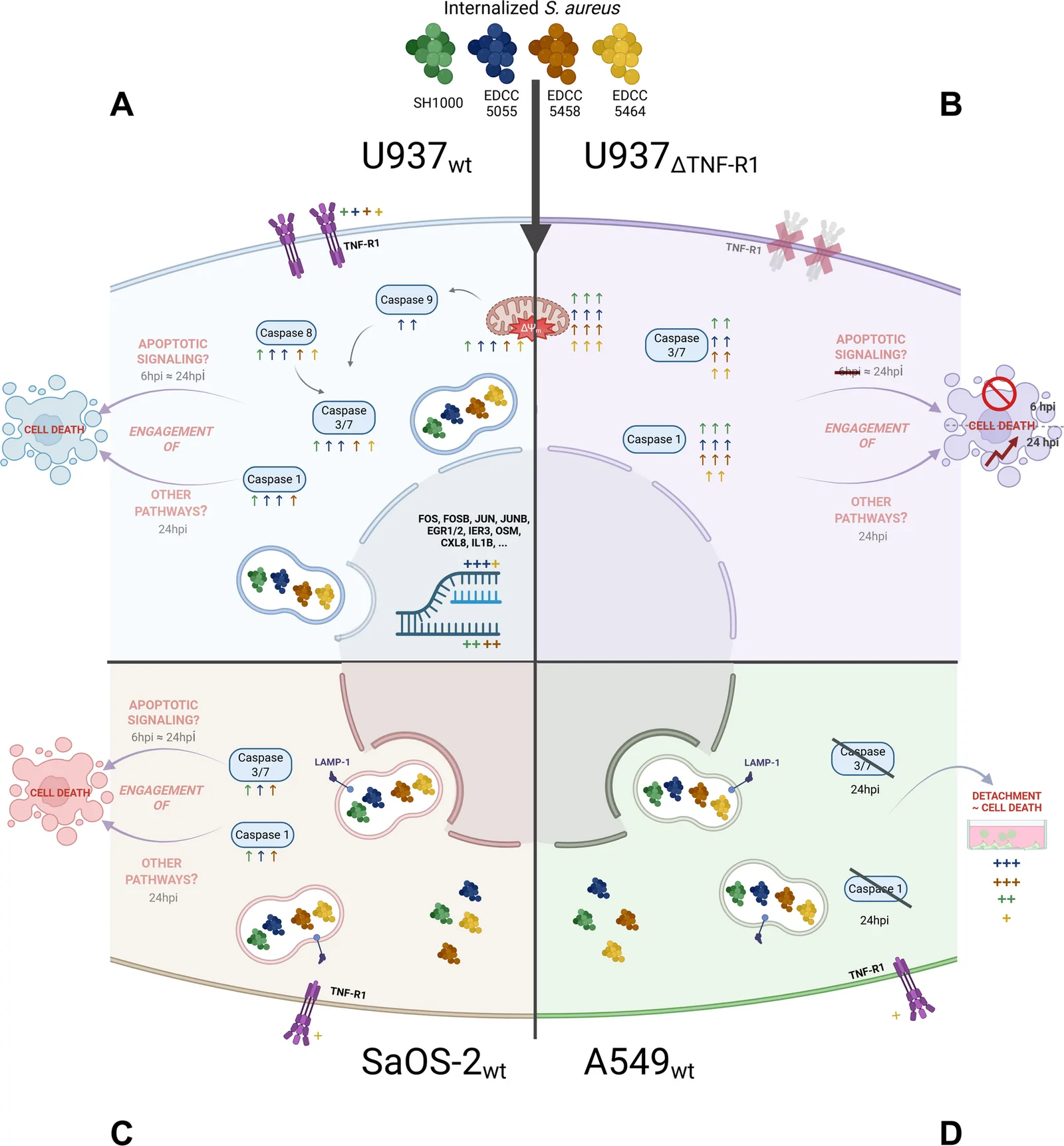

**Supplementary Information:**

The online version contains supplementary material available at https://doi.org/10.1186/s12964-026-03246-8.

## Abstract (plain language summary)

The bacterium *Staphylococcus aureus* (*S. aureus*) often lives harmlessly in humans but can cause severe or recurrent infections when the skin barrier is broken or when the immune system is weakened. A major reason for its persistence is its ability to hide inside human cells, where it is shielded from immune attacks and antibiotics. To effectively target such bacteria, it is crucial to understand that infections vary depending on both the bacterial strain and the infected tissue type. Many reasons behind these differences are still puzzling. We explored how different types of *S. aureus* (collected from different disease types) change how different human cells respond to infection. We focused on how each strain influences the way immune cells adjust their gene activity during infection, and how a receptor called TNF‑R1 is involved in managing cell death responses.

Bacteria are found in compartments meant to destroy them but frequently also in close association to the cell nucleus, an unexpected location that requires further investigation. All strains triggered a similar stress response but also distinct patterns differentially influencing inflammation, metabolism, and cell survival. A strain linked to chronic infection caused weaker responses, suggesting greater ability to remain undetected. The immune cells had greater presence of TNF-R1 on their surface when infected, and when they lacked this receptor entirely, they initially survived longer but later showed greater damage, indicating its key role in infection control. In lung and bone cells, these effects were less pronounced.

In conclusion,* S. aureus* uses various survival strategies that have heterogeneous impact on different host tissues. Some bacteria localized to unexpected compartments close to the cell’s control center inside all analyzed human cell types. Importantly, while the exact mechanisms are not yet clear, TNF-R1 influences host infection responses in the studied immune cells, highlighting that the interaction of bacterial programs and critical host factors must be carefully considered when developing targeted treatments.

**Supplementary Information:**

The online version contains supplementary material available at https://doi.org/10.1186/s12964-026-03246-8.

## Background

*Staphylococcus aureus* (*S. aureus*) asymptomatically colonizes up to one third of healthy individuals yet remains a major cause of community‑ and hospital‑acquired infections that range from superficial disease to severe pneumonia, endocarditis, osteomyelitis, and sepsis [1, 2]. Increasing evidence shows that a majority of *S. aureus* strains are proficient intracellular pathogens capable of invading, surviving, and persisting within diverse professional and non‑professional phagocytic cell types. These include phagocytosis by professional phagocytes such as neutrophils, monocytes and macrophages, as well as internalization by nonprofessional phagocytic epithelial and endothelial cells, keratinocytes, and osteoblasts [3–11]. This intracellular versatility is supported by high phenotypic plasticity and tightly regulated virulence programs, including agr, saeRS, sarA, or sigB [12, 13], enabling adaptation to distinct tissue niches, evasion of immune surveillance, and persistence despite or even promoted by antibiotic treatment with limited intracellular activity. Consequently, intracellular survival critically contributes to chronic and therapy-refractory infections [14, 15].

Infection outcomes, however, are shaped not only by strain‑specific virulence patterns but also by the host cell type, resulting in highly specialized host‒pathogen interactions. Professional phagocytes act as a first line of antimicrobial defense, but may act as “Trojan horses” during systemic *S. aureus* dissemination when bacterial clearance fails [4, 16–19]. In contrast, nonprofessional phagocytic cells may internalize bacteria less efficiently yet provide distinct intracellular niches that determine host and bacterial fate.

Once internalized, *S. aureus* disrupts phagolysosomal trafficking and maturation, which can be followed by escape into the cytosol and the modulation of cellular stress and inflammatory pathways. Manipulation of host cell death signaling is particularly important for whether intracellular bacteria are contained, eliminated, or are ultimately released in a manner that promotes inflammation and dissemination [20–22].

Central to this balance is the activation of the tumor necrosis factor-α (TNF)/TNF receptor 1 (TNF-R1) system, which coordinates inflammation, antimicrobial defense, and regulated cell death. However, excess TNF signaling can potentiate cell death to some extent, resulting in cellular demise and severe tissue damage [23]. The formation of signaling *complex I* on plasma membrane resident receptors triggers downstream activation of NF-κB and MAP kinases (MAPKs) to promote cell survival, proliferation, and activation of cytokine signaling, while re-organization of *complex I* to *complex IIa/b* results in the activation of apoptotic cell death cascades [23, 24]. When caspase-8 is inactive or absent, signaling shifts towards necroptosis [25]. In some cell types, full activation of TNF-mediated apoptosis requires endocytosis of *complex II* bearing TNF-R1 [26]. Indeed, several bacteria have evolved mechanisms to subvert this axis by inhibiting TNF-R1 internalization or endolysosomal fusion processes, thereby preventing lysosomal degradation and apoptosis to maintain their replicative niche [27–30].

Clinically, blockade of TNF signaling may attenuate excessive inflammation and severe tissue damage by overshooting immune responses during infection. However, anti-TNF treatment in autoimmune diseases increases susceptibility to and recurrence of infections by opportunistic and intracellular pathogens, including *S. aureus,* due to impaired TNF-dependent immune defenses necessary for adequate bacterial clearance [31–34]. Thus, balancing anti-inflammatory benefits with infection risk requires a precise understanding of TNF-R1–mediated control of intracellular *S. aureus* survival, host cell death modalities, and tissue-specific outcomes.

Despite extensive work on intracellular *S. aureus*, comparisons across strains and host cell types remain limited, complicating generalizable conclusions beyond the specific model used in each study [6, 9]. To address this, we systematically investigated the host responses of the human monocytic cell line U937 infected with the well-characterized *S. aureus* isolate SH1000 alongside three clinical strains of different, sepsis- and acute or chronic implant-associated bone infection backgrounds (EDCC 5055, EDCC 5458, and EDCC 5464) [35–38] and compared the infection outcomes with those of nonprofessional phagocytic lung-epithelial and osteoblast-like A549 and SaOS-2 cell lines.

All isolates were internalized into membrane-enclosed compartments, along with cytosolic and frequent perinuclear localization. Despite a conserved stress response across *S. aureus* isolates, each induced specific host transcriptional alterations in U937 monocytes, which is consistent with heterogeneous cytokine release and cytotoxicity. Host cell death involved isolate-dependent activation of different caspases consistent with apoptotic and inflammasome/caspase-1 signaling at different stages of infection in U937 and SaOS-2 cells, whereas A549 cells presented minimal caspase activation despite isolate-specific detachment and a greater fraction of Annexin V/7-AAD-positive cells.

Infection increased TNF-R1 surface expression in U937 cells, whereas receptor deficiency abolished early regulated cell death but exacerbated late cytotoxicity across isolates, suggesting a critical role for TNF-R1 in the finely controlled balance of regulated host defense and deleterious cytotoxicity. This effect was cell type-specific, and was less pronounced in A549 and SaOS-2 cells, which presented enhanced TNF-R1 surface expression only following infection with the chronic isolate EDCC 5464.

Together, these findings reveal pronounced isolate- and tissue-specific responses during intracellular *S. aureus* infection and identify TNF-R1 as a critical determinant of monocyte infection outcomes, emphasizing the need for tissue-directed therapeutic strategies and careful consideration of TNF-modulating treatments in the context of intracellular bacterial infections.

## Methods

### Human cell lines, bacterial strains and culture conditions:

U937_wt_/U937_ΔTNF-R1_ [39], A549_wt_/A549_ΔTNF-R1_, and SaOS-2_wt_/SaOS-2_ΔTNF-R1_ cells were cultivated in RPMI (Gibco), DMEM (Gibco), and McCoy’s (Capricorn Scientific), respectively, supplemented with 10% FBS (Gibco) and 1% sodium-pyruvate (Gibco) without antibiotics at 37 °C, 5% CO_2_.

*Staphylococcus aureus* (*S. aureus*) strains EDCC 5055, EDCC 5458, EDCC 5464, and SH1000 [35–38] (kindly provided by GK. Mannala and S. Mühlen) were grown in Luria–Bertani broth (LB) aerobically at 37 °C and 180 rpm overnight. The bacterial overnight cultures were adjusted to the stationary growth phase at an optical density at 600 nm (OD_600_) of 1.0–1.5 and incubated for at least 30 min at 37 °C and 180 rpm. To calculate the multiplicity of infection (MOI), the bacterial number was determined with an OD_600_ of 1 corresponding to 8 × 10^8^ colony-forming units (CFU) per ml.

#### TNF-R1 knockdown

For CRISPR-mediated knockdown, we used commercial Cas9 (IDT) loaded with crRNA:tracrRNA duplexes as recommended by the manufacturer’s protocol (Alt-R CRISPR-Cas9, IDT). Two TNF-R1 gene targets, predesigned crRNAs (Hs.Cas9.TNFRSF1A.1.AA: TAATGTATCGCTACCAACGG; Hs.Cas9.TNFRSF1A.1.AC: AGAGGTGCACGGTCCCATTG) were used to enhance knockout performance, as recommended [40]. Complexes were delivered by nucleofection (Amaxa), followed by Western blot screening.

### Intracellular infection assays

Unless stated otherwise, A549 and SaOS-2 cells were seeded at a density of 5 × 10^5^ cells/ml overnight, and U937 cells were adjusted to 1 × 10^6^ cells/ml prior to infection. The cells were infected with live or heat-killed (10 min, 95 °C) *S. aureus* at MOI 30 (all functional and quantitative assays) or MOI 100 (imaging, fractionation), followed by 1 h of coculture at 37 °C, 5% CO_2_ to allow for synchronized intracellular uptake. To remove extracellular bacteria, the cells were washed with PBS and resuspended in fresh medium containing gentamicin (G1397; Sigma-Aldrich) at 37.5 µg/ml to investigate infection-induced effects caused by only intracellular *S. aureus* (gentamicin protection assay). This relatively low gentamicin concentration was used because higher concentrations could penetrate the host cell membrane and kill *S. aureus* in the intracellular compartments [36] or affect the overall cell metabolism [41]. Efficient inhibition of bacterial growth by the concentration used was verified by multiple methods for each isolate, including inhibition zone testing and plating of gentamicin-treated cell supernatants containing extracellular bacteria that remained after washing at 1 h post infection (hpi), confirming the absence of bacterial growth.

### TNF-R1 surface expression assay

The cells were infected with viable or inactivated, CFSE-labeled or unlabeled *S. aureus* isolates according to the previously described intracellular infection assay. At 2 hpi, 5 × 10^5^ cells were sedimented, and staining was performed with a biotin-conjugated anti-CD120a (TNF-R1) antibody (#130–106–284, Miltenyi Biotec) along with Streptavidin-AlexaFluor 647 (1:100; S32357, Thermo Fisher Scientific) according to the manufacturer’s recommendations. The results were analyzed using Guava easyCyte (Cytek). Staining controls included: untreated cells without labeling, with CD120^+^/AF647^−^ or CD120^+^/AF647^−^, and infected cells without labeling.

### Cell death analysis

For each assay, untreated cells served as negative controls. For positive controls for apoptosis, the cells were primed with cycloheximide (2.5 µM; API-03, VWR) 30 min prior to stimulation with TNF (100 ng/mL; provided by D. Männel, UKR) or stimulated with nigericin (20 µM; Cay11437-5, Biomol) for caspase-1 activation indicating pyroptotic pathway activation. TNF-antagonists (10 µg/ml, anti-TNF Fab’, provided by D. Männel, UKR) were added 30 min prior to infection and the mixture was replenished after washing at 1 hpi. At the indicated time points, staining was performed following the manufacturer’s instructions, unless otherwise stated. Caspase-3/7 and multiple-caspase assays were measured using Guava MUSE (Cytek). Annexin-V, caspase-1, −8, and −9 assays using a Guava easyCyte (Cytek) and guavaSoft (4.5.25, Cytek) for data analysis.

#### Annexin V/7-AAD staining

In brief, 250 µl and 500 µl of carefully resuspended U937 and A549/SaOS-2 cells, respectively, were sedimented (500 × g, 5 min), washed twice with cell staining buffer (#420201, BioLegend), and resuspended in 20 µl staining mix (18 µl PBS, 1 µl FITC-Annexin V, 1 µl 7-amino-actinomycin D (7-AAD); #640922, BioLegend). After incubation for 20 min in the dark, 100 µl of binding buffer was added to the samples and then kept on ice prior to measurement.

#### Caspase assays

For Muse® Caspase-3/7 (#MCH100108, Cytek) and Multi-Caspase assays (#MCH100109, Cytek), 50 or 100 µl of carefully resuspended cells were sedimented (following detachment in case of adherent cells), washed with PBS (5 min, 500 × g), and the pellet was stained following the manufacturer’s recommendations. In brief, each pellet was resuspended in 50 µl 1 × assay buffer, 5 µl caspase-working solution was added (substrate diluted 1:8 in PBS for Caspase-3/−7 or 1:160 for Multi-Caspase), and incubated for 30 min under cell culture conditions, followed by incubation with 148 µl assay buffer and 2 µl 7-AAD in the dark for 5 min at room temperature.

To assess caspase-1 positive cells, the FAM-YVAD-FMK substrate (#13483, Biomol) was diluted 1:250 in 250 µL cell suspension with 5 µL 7-AAD (#420404, BioLegend), followed by 1 h incubation under cell culture conditions. Cell suspensions were washed once in PBS (5 min, 500 × g), resuspended in 100 µl PBS, and immediately analyzed. For Caspase-8 (FAM-LETD-FMK (656), #ICT099, BioRad) and Caspase-9 (FAM-LEHD-FMK (677), #ICT912, BioRad), 10 µl of caspase working solution (1:150 in PBS) were added to 290 µl of the cell suspension. After 60 min of incubation under cell culture conditions, cells were washed twice using 1 × Apoptosis Wash Buffer (AWB) and resuspended in 300 µl 1 × AWB. Thereafter, samples were incubated with 1,5 µl (0.5% v/v) of propidium iodide (PI) for 5 min in the dark at room temperature. All samples were kept on ice and analyzed immediately after staining.

As an essential indicator of cellular health, stress, and death, changes in mitochondrial trans-membrane potential (ΔΨ_m_) along with cell membrane permeabilization were monitored via the Muse® MitoPotential assay (#MCH100110, Cytek) following the manufacturer’s instructions. In brief, the MitoPotential working solution was prepared at 1:1,000 in 1 × assay Buffer, 95 µl was added to 100 µl of the cell suspension and incubated for 20 min under cell culture conditions. Following incubation with 5 µl of 7-AAD in the dark for 5 min at room temperature, samples were analyzed immediately.

#### SDS-PAGE and western blot

After the indicated time points, cells were sedimented and washed with PBS (5 min, 500 × g, 4 °C). The cell pellets were lysed in 30 µl modified RIPA buffer (50 mM TRIS–HCl [pH 7.5], 150 mM NaCl, 1% NP-40, 1% Triton X-100, 1 mM EDTA, 0.25% Na-deoxycholate), containing a protease inhibitor cocktail (4693124001, Sigma-Aldrich), followed by 20 s sonication (cooled to 4 °C) and 5 min sedimentation at 1500 × g at 4 °C. The supernatant was used for BCA protein quantification (Pierce, #23225). Samples (20 µg) were prepared immediately before SDS-PAGE using 12.5% or 10% PAA gels. Proteins were blotted onto a PVDF membrane (200T.1, Carl-Roth). The membranes were blocked with 5% skimmed milk in TBS-T and incubated overnight with the primary antibodies, diluted 1:1,000 in 5% skimmed milk. The peroxidase-conjugated secondary antibodies were incubated for 1 h, diluted 1:10,000 in TBS-T. Blots were imaged using ECL (RPN2232, Cytiva) and the ImageQuant800 device (Cytiva). Primary antibodies: PARP-1 (cs #9542S), cleaved caspase-3 (#9661S, Cell Signaling), caspase-3 (#9668S, Cell Signaling), Actin-HRP (HRP-60008, Proteintech). Secondary antibody: anti-rabbit-HRP (111–035–144, Jackson ImmunoResearch).

#### xCELLigence real time cell analysis (RTCA)

To analyze both the extent and kinetics of cell death of adherent cells, xCELLigence Real-time cell analysis (RTCA, OMNI Life Science) assay was performed following the manufacturer’s instructions. In brief, A549 or SaOS-2 cells were seeded overnight at 8000 cells per well in 100 µl medium after a blank measurement using 50 µl of the corresponding cell culture medium. The attached cells were then infected in duplicates with the corresponding *S. aureus* isolates at MOI 30 and co-cultured for 1 h. Along with a duplicate of entirely untreated or only gentamicin-treated cells, both uninfected and infected cells were then washed three times in and supplemented with fresh cell culture medium containing 37.5 µg/ml gentamicin (G1397; Sigma-Aldrich). The detachment of cells based on cellular impedance (indicating cell death) was monitored every 15 min for at least 48 hpi.

### Luminex multiplex ELISA®

To monitor the release of selected cytokines, 1 × 10^6^ U937 cells or 5 × 10^5^ A549 cells were infected with *S. aureus* at MOI 30. At 4 hpi, cells were centrifuged at 14,000 xg for 10 min. A 48-plex Luminex Multiplex ELISA (Bio-Rad Laboratories, #12007283) was performed using the supernatants according to the manufacturer''s protocol [42], using the Bio-Plex 200 system with HTF (#171000205, Bio-Rad Laboratories). The assay was subsequently repeated via a customized 8-plex Luminex Multiplex ELISA (IL-6, IL-8 (CXCL8), MCP-1 (CCL2), MIF, MIP-1α (CCL3), RANTES (CCL5), SCGFβ, TNF-α) (#PPX-08, Thermo Fisher) in all three cell lines.

### Labeling of bacteria

For CFSE-staining, bacteria were labeled with 5 µM CFSE/PBS for 1 h, shaking at 37 °C, followed by three washing steps (10,000 × g, 10 min) in PBS. The number of labeled bacteria was determined photometrically at OD_600_ and used for infection. For Lipobiotin/Streptavidin-labeling we followed [43]. The bacterial suspension was centrifuged (10 min, 10,000 × g) and the sediment was resuspended in 300 µl PBS. 5 µg/ml of lipobiotin (L2070; EMC microcollections) was added, together 10 µl of Streptavidin-magnetic nanobeads (130–048–102; Miltenyi Biotec). After incubation at 37 °C for 1 h, bacteria were washed once in PBS (10 min, 10,000 × g), then resuspended in 500 µl PBS. This labeling strategy was used merely for TEM samples to verify internalization of labeled bacteria, relevant for follow-up studies. Comparable uptake rates of labeled vs. unlabeled bacteria were confirmed by CFU counts and flow cytometry (Fig. S3 Li,ii).

### Subcellular fractionation

A549, SaOS-2 and U937 cells were infected with *S. aureus* isolates EDCC 5055 or EDCC 5464 at MOI 100 via a gentamicin protection assay. After infection, the cells were washed with PBS, resuspended in 1 ml cold cell culture medium, and subjected to subcellular fractionation by lysis gradient centrifugation in 5 ml polystyrene tubes with rounded bottoms [44]. The discontinuous iodixanol gradient comprised a cell wash layer (5% iodixanol in PBS), a lysis layer (10% iodixanol supplemented with 4 µg/mL Coomassie Brilliant Blue, 5 µg/ml crystal violet, 1 × protease inhibitor and 0.5% Nonidet P40 in PBS), a nucleus wash layer (20% iodixanol supplemented with 1 × protease inhibitor in PBS), and a floating layer (35% iodixanol in PBS). A total of 1 ml of cell suspension was loaded onto the cell wash layer. The gradients were centrifuged at 1,000 × g for 10 min at 4 °C in a swinging-bucket rotor. For the nuclear fraction, 300 µl were collected (banded nuclei), 500 µl PBS were added, followed by centrifugation for 30 min at 2,000 × g and 4 °C. This step was repeated at least once and, if required, the fraction was further diluted with PBS until efficient pelleting was achieved. The supernatant was subsequently discarded and the pellet was washed with 1 ml nuclear washing buffer (NWB: 10 mM HEPES, 10 mM KCl, 1.5 mM MgCl2, 1 mM EDTA, 0.5 mM DTT, 1 × protease inhibitor (Roche complete mini)) for 15 min at 2,000 × g, 4 °C. After removing the supernatant, the pellet was resuspended in 200 µl PBS. Bacterial viability was confirmed by plating 10 µl of the nuclear suspension onto LB agar. PBS was then added to the suspension to a final volume of 1 ml, centrifuged for 15 min at 2,000 × g, 4 °C, and the resulting pellet was lysed in 17 µl mod. RIPA/PIC. For the cytosolic fraction, 600 µl of the cytosolic layer were collected and concentrated using VivaSpin 6 columns (10 kDa cutoff) by centrifugation at 3,300 × g for 25 min at 4 °C to a final volume of approximately 200 µl. Bacterial viability was confirmed by plating 10 µl of the cytosolic suspension onto LB agar. The concentrated cytosolic fraction was then mixed with modified RIPA/PIC at a ratio of 3:1 (sample:RIPA/PIC). Whole-cell lysates, cytosolic fractions and nuclear fractions were subsequently analyzed by Western blot (see above). The primary antibodies used were as follows (1:1,000): pan-*S. aureus* (#PA1-73173, Thermo Fisher Scientific), nuclear markers: Lamin B1, #17416, Cell Signaling Technology; Histone H3, #9715S, Cell Signaling Technology), cytosolic markers: HSP90β (#37–9400, Invitrogen), GAPDH (HRP-60004, Proteintech), α-tubulin (HRP-66031, Proteintech).

### Transmission electron microscopy (TEM)

Adherent cells were seeded on Corning™ BioCoat™ fibronectin-coated coverslips (Fisher Scientific, #08–774-386) one day, and U937 suspension cells were seeded directly prior to infection in crowded conditions in a 1.5 ml tube. Cells were infected with either two (A549, SaOS-2, *n* = 2) or four (U937, *n* = 1) of the *S. aureus* isolates at MOI 100, respectively, which were labeled with lipobiotin and streptavidin-conjugated magnetic nanobeads (130–048–102; Miltenyi Biotec), which will be used for targeted organelle purification in future investigations.

Following a washing step and gentamicin treatment at 1 hpi, the cells were fixed at 2 hpi in 2% glutaraldehyde (Serva Electrophoresis) buffered with 100 mM sodium cacodylate, pH 7.4 for 1 h at room temperature. Afterwards, the suspended cells were coated with 4% low melting agarose (840302; Biozym), dissolved at 37 °C in 100 mM sodium cacodylate and then cooled for 35 min on ice. After several washing steps with 100 mM sodium cacodylate, all the cell lines were further fixed via 0.5% osmium tetroxide (Science Services) and 0.5% uranyl acetate was added for optimal contrast. Both treatments were performed for 30 min at 4 °C. The cells were dehydrated in an ascending series of ethanol and finally acetone on ice. For equilibration, the samples were treated overnight at 4 °C in a 1:1 solution of Epon (Fluka) and acetone, followed by embedding with pure Epon for 2 h at 30 °C and polymerization for 2 d at 60 °C. For adherent cell samples, cover slips were removed without damaging the embedded cells by alternately exposing them to liquid nitrogen and heated water (60 °C). Ultrathin sections were cut with a diamond knife (Diatome) on an ultramicrotome EM UC7 (Leica) and collected on a hexagonal 200 mesh copper grid (Plano). The electron micrographs were taken at 80 kV on a Zeiss 902 or at 60 kV on a Zeiss EM10a transmission electron microscope (Zeiss). Several cells of different ultrathin sections were captured at different magnitudes and TEM pictures were optimized for brightness and contrast using ImageJ [45].

### Serial block-face scanning electron microscopy (SBF-SEM)

Cultured cells grown on 8-well chambered coverslips (IBIDI) were fixed in 0.1 M cacodylate buffer (pH 7.4, Science Services, #E11652) containing 2.5% glutaraldehyde (Science Services, #E16216). Samples were subsequently processed following a modified NCMIR rOTO-post-fixation protocol [46], which enhances contrast and confers resistance to electron beam damage during serial imaging of EPON resin-embedded specimens.

### Imaging flow cytometry

A total of 1 × 10^6^ U937 cells were infected with CFSE-labeled bacteria for 2 h at MOI 100. At 1 hpi, gentamicin was added to kill extracellular bacteria. 5 min before the end of the infection period, Cell Mask® deep red membrane stain (C10046; Thermo Fisher) was added, diluted 1:25,000 in PBS, and incubated for 5 min, followed by washing twice in cold PBS. Cells were then fixed in 4% PFA/PBS for 15 min on ice, followed by washing in 1 ml cold PBS. The cells were stored in the dark and cooled until imaging using ImageStream MKII (Cytek). A total of 10,000 images per condition were acquired at 60 × using the Inspire software, capturing channels Ch01 (Brightfield), Ch02 (CFSE), and Ch11 (CellMask deep red). Images were analyzed using Ideas software (V6.2.65): Cells were gated as follows: in-focus/single cells/double positive. Then, composites of Ch02/Ch11 were generated.

### SIM imaging

SIM was performed on a Zeiss Elyra 7 equipped with a C-Apochromat 63x/1.4 NA oil-immersion objective and following laser for excitation: high-reflectivity (HR) Diode 405 nm (50 mW), HR Diode 488 nm (500 mW), HR diode-pumped solid state 561 nm (500 mW), and HR Diode 642 nm (500 mW). Emission light was passed through these dichroic filter sets: BP420-480 + LP655 for the DAPI and Alexa Fluor 647 channels and BP495-525 + LP655 for the Alexa Fluor 488 and CFSE channels. Emission light was further split by a SBS LP 560 beam splitter and detected on two PCO Edge 4.2M sCMOS camera sensors. For 3D-SIM imaging appropriate grid-settings, laser lines and filter combinations were chosen following the suggested settings by the ZEN black microscope control software. Z-stacks were acquired using the Z-step interval optimized for “3D leap mode” as defined by ZEN, with 13 phase shifts recorded per z-plane. 3D-SIM reconstruction was performed in ZEN black using the standard SIM processing preset for fixed samples. Chromatic aberration was corrected by imaging a multicolor bead sample (T14792, Thermo Fisher) using identical imaging and processing settings. A channel alignment matrix was then generated in ZEN black and applied during the SIM processing of the sample images.

### Post-processing of SIM images

SIM images were processed in Imaris 11.0.1 (Oxford instruments) using the spot detection and surface generation functions. First, nuclei were segmented using the surface generation tool with the integrated machine-learning–based training workflow. Nuclear surfaces were subsequently filtered by voxel count to remove segmentation artifacts and nuclei not fully contained within the field of view. Surfaces for LAMP-1 or CFSE were similarly segmented using the surface function. LAMP-1 surfaces were filtered to include only surfaces with > 2000 voxels, CFSE surfaces were restricted to > 0.35 µm^3^. Bacterial DNA stained by DAPI was detected using the spot detection function with an estimated diameter of 0.42 µm and background subtraction enabled. Detected spots were filtered by Imaris “Quality” parameter and further curated with a machine-learning assisted step to exclude false positive detections from high heterochromatin densities within the cellular nucleus. Correct spot detection based on DNA staining (“all spots”) and appropriate spot size selection was verified using additional CFSE-labeled bacteria (Fig. S3Kii). The shortest distances from detected bacteria to the nucleus and lysosome surface (LAMP-1-stained) were extracted to quantify bacterial localization and LAMP-1-spot proximity. For plotting bacterial distances to the nucleus in CFSE-stained samples, only bacterial DNA spots with a maximal distance of 0.3 µm to the closest bacterium-specific CFSE surfaces were included for analysis (Fig. S3Kiii).

### Quantification of intracellular bacteria – colony-forming units

Cells were infected as described previously. At the indicated time points, cells were washed with PBS (500 × g, 5 min for U937 cells) to remove bacteria that had been released from the host cells. Thereafter, cells were lysed using 100 µl or 500 µl of 1% Triton X-100 (in ddH_2_O) for suspension cells and adherent cells, respectively, and incubated at 37 °C for 10 min. Following thorough vortexing and resuspension, the lysate was serially diluted, 10 µL of the dilution were plated on LB-agar plates and incubated overnight. CFU were calculated as follows:

$$\begin{aligned}\mathrm{CFU} \left(\mathrm{per}\;\mathrm{lysate}\right)&=\frac{Number\;of\;Colonies\;x\;Dilution\;Factor}{Plated\;Volume\;[mL]}\\& \quad\times \frac{1}{Total\;Lysate\;Volume\;[mL]}\end{aligned}$$

### RNA sequencing and data analysis

To screen for differential gene expression upon infection, U937 cells were infected with four different isolates of *S. aureus* or, for comparison, vancomycin-resistant *E. faecium* for 4 or 6 h, respectively, or left untreated. RNA was isolated using RNAplus kit (Qiagen). Library preparation and RNAseq were carried out as described in the Illumina “Stranded mRNA Prep Ligation” Reference Guide, the Illumina NextSeq 2000 Sequencing System Guide (Illumina), and the KAPA Library Quantification Kit—Illumina/ABI Prism (Roche Sequencing Solutions, Inc., Pleasanton, CA, USA). 200 ng of total RNA was used for purifying the poly-A-containing mRNA molecules using oligo(dT) magnetic beads. Following purification, the mRNA was fragmented to an average insert size of 200–400 bases using divalent cations under elevated temperature (94 °C for 8 min). Next, the cleaved RNA fragments were reverse transcribed into first-strand complementary DNA (cDNA) using reverse transcriptase and random hexamer primers. Thereby, Actinomycin D was added to allow RNA-dependent synthesis and to improve strand specificity by preventing spurious DNA-dependent synthesis. Blunt-ended second-strand cDNA was synthesized using DNA Polymerase I, RNase H, and dUTP nucleotides. The incorporation of dUTP, in place of dTTP, quenches the second strand during the later PCR amplification, as the polymerase does not incorporate past this nucleotide. The resulting cDNA fragments were adenylated at the 3'' ends, and the pre-index anchors were ligated. Finally, DNA libraries were created using a 13 cycles PCR to selectively amplify the anchor-ligated DNA fragments and to add the unique dual indexing (i7 and I5) adapters. The libraries were bead-purified twice and quantified using the KAPA Library Quantification Kit. Equimolar amounts of each library were sequenced on an Illumina NextSeq 2000 instrument controlled by the NextSeq 2000 Control Software (NCS) v1.7.0.45751, using 65 read cycles on a P3 Flow Cell with the dual index, single-read (SR) run parameters and the dark cycle recipe. Image analysis and base calling were done by the real-time analysis software (RTA) v4.12.2. The resulting.cbcl files were converted into fastq files using bcl2fastq v2.20 software. Library preparation and mRNAseq were performed at the Genomics Core Facility “KFB—Center of Excellence for Fluorescent Bioanalytics” (University of Regensburg, Regensburg, Germany; www.kfb-regensburg.de).

The raw data was processed at the Core Unit Systems Medicine, University of Würzburg (Würzburg, Germany; https://www.med.uni-wuerzburg.de/cu/sysmed/). To assure high sequence quality, Illumina reads were quality- and adapter-trimmed via Cutadapt [47] v2.5 using a cutoff Phred score of 20 in NextSeq mode, and reads without any remaining bases were discarded (parameters: –nextseq-trim = 20 -m 1 -a ACTGTCTCTTATACACATCT). Processed reads were subsequently mapped to the human genome (NCBI RefSeq assembly GCF_000001405.40/GRCh38.p14, primary assembly and mitochondrion) using STAR [48] v2.7.2b with default parameters but including transcript annotations from RefSeq annotation version RS_2023_03 for GRCh38.p14. This annotation was also used to generate read counts on exon level summarized for each gene via featureCounts v1.6.4 from the Subread package [49]. Multi-mapping and multi-overlapping reads were counted strand-specific and reversely stranded with a fractional count for each alignment and overlapping feature (parameters: -s 2 -t exon -M -O –fraction). The count output was utilized to identify differentially expressed genes using DESeq2 [50] v1.24.0. Read counts were normalized by DESeq2 and fold-change shrinkage was conducted by setting the parameter betaPrior to TRUE. Separate DESeq2 analyses were conducted for comparisons within *S. aureus* and *E. faecium* infection data, both including the uninfected control. Results shown here focus on *S. aureus*-infected cells.

Principal component analysis (PCA) was performed by applying variance-stabilizing transformation (vst) to normalized read counts via DESeq2. The 1000 most variable genes (after removal of zero/NA-variance features) across all samples were selected and scaled prior to PCA computation using R (*prcomp*, stats package; R version 4.5.1, RStudio 2025.5.1.513). Samples were grouped by condition (9 levels: SH1000, EDCC 5055, EDCC 5458, EDCC 5464, untreated, and four *E. faecium* strains VRE 23 × 1608, VRE 23 × 1609, VRE 7282, and VRE 8170) and PCA scores were plotted using ggplot2 (version 3.5.2).

Gene lists used for all downstream visualization and functional analyses were defined as protein-coding, significantly regulated (adjusted p-value after Benjamini–Hochberg correction (padj) < 0.05), and at least two-fold up- or downregulated (|log2FC|≥ 1) relative to uninfected control cells or between cells infected with different *S. aureus* isolates, as applicable. Full STRING networks (https://string-db.org/) were generated at medium confidence (0.400), with edges indicating confidence and disconnected nodes removed. Heat maps of selected genes were created using GraphPad Prism 10.6.1.

Functional enrichment of biological pathways that are present in these gene lists more than expected by chance was performed with g:Profiler [51] (version e113_eg59_p19_f6a03c19). Only annotated genes were submitted as ordered queries using the g:SCS multiple-testing correction and a significance threshold of 0.05, allowing the analysis to account for the ranked structure of the input lists without imposing additional arbitrary cutoffs. Gene Ontology Biological Process (GO:BP) terms were used for all downstream analyses. For ordered queries, the adjusted values reported by g:Profiler do not represent classical *P*-values but are to be interpreted as enrichment scores (adj_score) that reflect the concentration of term-associated genes at the top of the ranked list rather than random distribution [52].

To identify non-redundant groups of related GO:BP terms, enriched up- and downregulated pathways were imported separately for each condition into R (R version 4.5.1, RStudio 2025.5.1.513) and processed with *tidyverse* (version 2.0.0) and *openxlsx* (version 4.2.8). For every term, the associated gene set was represented as a vector of gene symbols derived from the g:Profiler “intersections” column. Gene-set similarity was quantified as the Jaccard coefficient (intersection/union of member genes), as commonly applied in gene-set clustering tools such as EnrichmentMap and GOMCL [53–55]. Hierarchical clustering (*hclust*, *stat* package) was performed based on a Jaccard distance matrix (1 − Jaccard coefficient) between pathways using average linkage. To obtain interpretable clusters with comparable granularity across isolates, dendrograms were cut at height *h* = 0.5 (*cutree*), corresponding to a minimum within-cluster Jaccard similarity of 0.5, consistent with conservative Jaccard thresholds recommended for pathway network construction. Small or single-pathway clusters were retained, as the goal was to preserve biologically specific terms rather than impose arbitrary minimum cluster sizes. Each cluster was named manually according to the driver term. The minimum adjusted enrichment score (min_adj_score) among its member pathways and the total number of genes of each cluster were extracted and visualized as bubble plots in R using *ggplot2 (version 3.5.2)*, with bubble size encoding gene count per cluster and bubble color representing − log10 of the cluster-level min_adj_score, similar to standard enrichment bubble plots. R scripts for pathway clustering and visualization are provided in Supplementary Data S1.

### Sequencing, assembly, and annotation of S. aureus genomes

Four isolates were sequenced using both short-read Illumina and long-read Oxford Nanopore technologies. Illumina sequencing was performed on a NextSeq platform using Illumina DNA Prep chemistry (mid-output, 300 cycles, paired-end 2 × 149 bp). Long-read sequencing was conducted on a GridION device using R10.4.1 flow cells and the Native Barcoding Kit 96 v14 (SQK-NBD114.96). Hybrid genome assemblies were generated from the corresponding FASTQ files using MiLongA version 1.0.3 [56]. For assembly generation and quality evaluation, only the components relevant for Flye-based assembly were applied, namely Flye v2.9.4-b1799 for long-read assembly, minimap2 v2.28-r1209 for read mapping, and CheckM v1.2.2 for genome completeness and contamination assessment. The Flye assemblies were selected for all downstream analyses. The resulting genome assemblies were annotated using the NCBI Prokaryotic Genome Annotation Pipeline (PGAP, release 2023–10-03.build7061) [57] and are available in Supplementary File S14.

To compare the genomes between the different isolates (Supplementary File S2), the assembled data were annotated using Prokka (1.14.6; [58]) and comparison tables were generatedviag ROARY (3.13.0; [59]).

### Proteomic profiling of* S. aureus *at the steady-state

#### Filter aided sample preparation (FASP)

For the proteome analysis, samples were prepared using a modified filter-aided sample preparation (FASP) as detailed before [60, 61]. In brief, *S. aureus* isolates were lysed adding 700 µL of lysis buffer containing 7 M urea, 2 M thiourea, 5 mM dithiothreitol (DTT), 2% (w/v) CHAPS. Lysis was promoted by sonication using a Branson sonifier at output 5 for 1 min, followed by centrifugation at 10,000 × g for 10 min to pellet remaining bacterial cells.

The protein concentration of the supernatants was determined using the Pierce 660 nm protein assay (Thermo Fisher Scientific) following the manufacturer´s protocol. Proteins (corresponding to 20 µg) were transferred onto spin filter columns (Micron-30 Centrifugal Filters; Merck Millipore). The samples were subsequently washed three times with a buffer containing 8 M urea. After reduction and alkylation by DTT and IAA, excess IAA was quenched with DTT, and the membrane washed three times with 50 mM NH_4_HCO_3_. Afterwards, proteins were digested overnight at 37 °C with trypsin (Trypsin Gold, Promega) using an enzyme-to-protein ratio of 1:50 (w/w). After digestion, peptides were recovered by centrifugation and two additional washes with 50 mM NH_4_HCO_3_. The combined flow-throughs were acidified with TFA to a final concentration of 1% (v/v) TFA and lyophilized. Purified peptides were reconstituted in 0.1% (v/v) FA for LC–MS analysis.

#### Liquid-chromatography mass spectrometry (LC–MS)

Proteome samples were analyzed using a nanoElute LC system (Bruker Corporation) coupled online to a timsTOF HT mass spectrometer (Bruker Corporation, Billerica, MA, USA). Peptides (corresponding to 200 ng) were separated at 400 nl/min using a reversed phase C18 column (Aurora ULTIMATE UHPLC emitter column, 25 cm × 75 µm 1.7 µm, IonOpticks), which was heated to 50°C. Peptides were loaded onto the column in direct injection mode at 600 bar. Mobile phase A was 0.1% FA (v/v) in water and mobile phase B 0.1% FA (v/v) in ACN. Peptides were separated running a linear gradient from 2 to 37% mobile phase B over 39 min. Afterwards, column was rinsed for 5 min at 95% B. Eluting peptides were analyzed in positive mode ESI–MS using parallel accumulation serial fragmentation enhanced data-independent acquisition (diaPASEF) [62]. The dual TIMS was operated at a fixed duty cycle close to 100% using equal accumulation and ramp times of 100 ms each spanning a mobility range from 1/K_0_ = 0.6 Vs cm^−2^ to 1.6 Vs cm^−2^. We defined 36 × 25 Th isolation windows from *m/z* 300 to 1,165 resulting in 2–3 diaPASEF scans per TIMS cycle and an overall cycle time of 1.7 s. The collision energy was ramped linearly as a function of the mobility from 59 eV at 1/K_0_ = 1.3 Vs cm^−2^ to 20 eV at 1/K_0_ = 0.85 Vs cm^−2^. The samples were measured in triplicate.

#### Raw data processing

MS raw data were processed separately for each isolate using DIA-NN (version 2.2.0) [63] applying the default settings for library-free database search. Custom databases were compiled using isolate transcript libraries as described above as well as a list of common contaminants. Trypsin was set as the protease for in-silico library generation and peptide identification, allowing one missed cleavage. Carbamidomethylation was set as a fixed modification and maximum number of variable modifications was set to zero. Peptide length ranged between 7–30 amino acids. The precursor m/z range was set to 300–1,800 and the product ion m/z range to 200–1,800. “QuantUMS (high precision)” mode was applied as quantification strategy with RT-dependent normalization. MS1 and MS2 mass accuracies were both set to 15 ppm and scan window size was automatically optimized using the build in algorithm of DIA-NN. Peptides precursor FDRs were controlled below 1%.

Proteins had to be identified by at least two peptides to be included in the final dataset.

### Data availability

RNA-seq data are available at https://www.ncbi.nlm.nih.gov/geo/query/acc.cgi?acc=GSE328149.

The mass spectrometry proteomics data have been deposited to the ProteomeXchange Consortium (http://proteomecentral.proteomexchange.org) via the jPOST partner repository [64] with the dataset identifiers PXD073241 (ProteomeXchange) and JPST004317 (jPOST). To review the data, go to: https://repository.jpostdb.org/preview/818370539696e370e1de6c with the access key: 2097.

### Statistical analysis

Statistical analysis was performed via GraphPad Prism 10.6.1 software (GraphPad Software Inc.). Normality and Long-normality Tests were performed using Shapiro–Wilk test. One- or Two-Way ANOVA with Tukey’s or Kruskal–Wallis correction for multiple comparisons were performed for testing of multiple groups, where appropriate. Comparisons were made for infected vs. uninfected control cells, between cells infected with different isolates or between cell types (*wild-type* versus *knockdown* or ± bc, ± TNF-Fab’), as indicated respectively. A *p* value of < 0.05 was considered statistically significant.

## Results

### *S. aureus* isolates are internalized by U937, A549, and SaOS-2 cells, primarily residing in bacteria-containing vacuoles, alongside cytosolic and nucleo-proximal association

Prior to internalization, we compared the *S. aureus* isolates SH1000, EDCC 5055, EDCC 5458, and EDCC 5464 [35–37]. Genome sequencing and steady‑state proteomics (1h culture in cell culture medium) revealed a shared core virulence repertoire across isolates prior to host cell contact (Table 1; Supplementary File S2), including major adhesins (FnBPA, ClfA, ClfB), pore‑forming toxins (α-hemolysin, LukGH), EsxA, staphopain A, or the regulators SrrA and SaeS. EDCC 5464 lacked detectable PSM‑α/β/δ and several Spl proteases, consistent with reduced lytic activity and a persistent phenotype, while EDCC 5055 uniquely expressed MecA.

**Table 1:** *S. aureus* steady-state expression of selected proteins prior to infection of host cells present with at least two peptides. Proteins expressed by all four isolates are highlighted (bold)

Protein Description	SH1000	EDCC 5055	EDCC 5458	EDCC 5464
PBP2a family beta-lactam-resistant peptidoglycan transpeptidase MecA		X		
penicillin-binding protein		X		
penicillin-binding protein 2		X	X	
beta-lactam sensor/signal transducer BlaRx		X		X
penicillin-hydrolyzing class A beta-lactamase BlaZ		X		X
**fibronectin-binding protein FnbA**	X	X	X	X
fibronectin-binding protein FnbB	X	X		X
**MSCRAMM family adhesin clumping factor ClfA**	X	X	X	X
**MSCRAMM family adhesin clumping factor ClfB**	X	X	X	X
**WXGx family type VII secretion effector EsxA**	X	X	X	X
**cysteine protease staphopain A**	X	X	X	X
**alpha-hemolysin**	X	X	X	X
bi-component gamma-hemolysin HlgAB subunit A			X	X
bi-component gamma-hemolysin HlgAB/HlgCB subunit B	X	X	X	X
bi-component gamma-hemolysin HlgCB subunit C	X	X	X	
staphylococcal enterotoxin type A		X		
staphylococcal enterotoxin type R		X		
staphylococcal protein A				X
**bi-component leukocidin LukGH subunit G**	X	X	X	X
**bi-component leukocidin LukGH subunit H**	X	X	X	X
**bifunctional autolysin**	X	X	X	X
phenol-soluble modulin PSM-alpha-4	X	X	X	
phenol-soluble modulin PSM-alpha-x			X	
beta-class phenol-soluble modulin	X	X	X	
delta-lysin family phenol-soluble modulin	X		X	
serine protease SplB	X	X		
serine protease SplD		X		
serine protease SplE		X		
serine protease SplF		X		
Fur family transcriptional regulator		X		
**staphylobilin-forming heme oxygenase IsdI**	X	X	X	X
LPXTG-anchored heme-scavenging protein IsdA	X	X	X	
von Willebrand factor binding protein Vwb		X	X	X
**two-component system response regulator SrrA**	X	X	X	X
**two-component system sensor histidine kinase SaeS**	X	X	X	X
HTH-type transcriptional regulator SarS			X	X

**Fig. 1:**
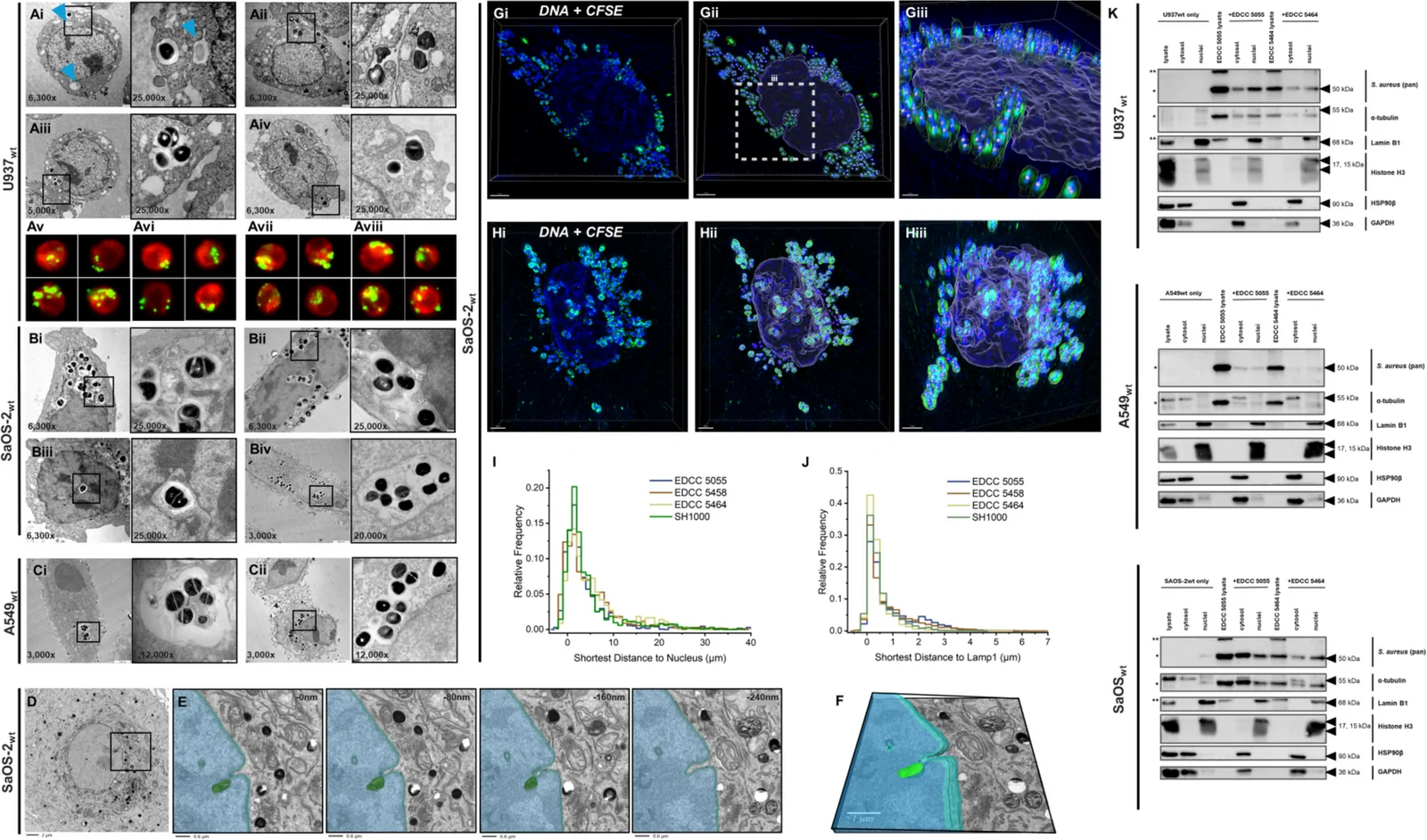
*S. aureus* isolates reside intracellularly in membrane-enclosed compartments, in the cytosol and associated with the host cell nucleus. Each cell line was infected with *S. aureus* at MOI 100 using a gentamicin protection assay and analyzed at 2 hpi. Transmission electron microscopy of (**A**) U937, (**B**) SaOS-2, and (**C**) A549 cells infected with (**Ai**) SH1000, **Aii**, **Bi**, **Biii**, **Ci** EDCC 5055, **Aiii** EDCC 5458, and **Aiv**, **Bii**, **Biv**, **Cii** EDCC 5464. Representative images at two different magnifications (black box, 3,000x-85,000 × as indicated) for *n* = 1 (U937) or *n* = 2 (A549, SaOS-2) independent experiments, each with n ≥ 10 analyzed cells. Blue arrows indicate bacteria of reduced electron-density, consistent with signs of degradation. **Av**-**viii** Imaging flow cytometry analysis shows CFSE-labeled bacteria (green) inside U937 host cells (red: CellMask deep red). **Av** SH1000, **Avi** EDCC 5055, **Avii** EDCC 5458, **Aviii** EDCC 5464. **D** SBF-SEM analysis of EDCC 5055-infected SaOS-2 cells shows nucleo-proximal localization lacking a surrounding vesicle. **E** Zoom-in of boxed region in D as pseudo-coloured sections of a total of 240 nm and **F** their 3D-reconstruction. **G**, **H** Maximum intensity projection of SaOS-2 cells infected with CFSE-labeled (**Gi**) EDCC 5055 or (**Hi**) EDCC 5464 with (**Gii**, **Hii**) zoomed-in or side view imaged by SIM (DNA is shown in blue, CFSE in green) showing nuclear association and invagination of the nuclear envelope. Surfaces and spots generated after image analysis in Imaris (11.0.1). Surfaces defining the nucleus are visualized by a blue outline, spot distance to CFSE-surface < 300nm: white, > 300nm: red. **I** Histogram of shortest distances between identified bacterial DNA-spots and the surface generated around the nucleus (bin size 1 µm; 8 images per strain; 1515 ± 160 (mean ± SD) detected spots per strain). **J** Histogram of shortest distances between identified bacteria spots and the surface generated around LAMP1 signals (Images see Fig. S3; bin size 0.25 µm; 8 images per strain; 1515 ± 160 (mean ± SD) detected spots per strain). **K** Nuclear fractionation of U937, A549, and SaOS-2 cells showing: whole-cell lysate, cytosolic, and nuclear fractions of uninfected, EDCC 5055- and EDCC 5464-infected cells, and bacterial lysates. Western blot analysis shows: pan-*S. aureus* to assess distribution across subcellular fractions, cytosolic markers (α-tubulin, HSP90β and GAPDH). Nuclear markers (Lamin B1 and Histone H3). Arrowheads indicate the expected molecular weights of the proteins. Asterisks (*; **) indicate pan-*S. aureus* signals upon re-probing blot membranes. Representative results of n ≥ 3 independent experiments

Bacterial uptake by U937 cells was verified by transmission electron microscopy (TEM; Fig. 1 Ai-Aiv) and imaging flow cytometry (Fig. 1 Av-Aviii), and by TEM for SaOS-2 and A549 cells (Fig. 1Bi-Cii). At 2 hpi, *S. aureus* was internalized and primarily resided within membrane-enclosed compartments of varying morphology (size, wide or tightly packed, reflecting different maturation states [65]), and variable numbers of engulfed bacteria were observed. Most vacuoles contained bacteria displaying division septa, consistent with ongoing replication, whereas changes in electron-density of single bacterial cells, particularly in U937 cells, might indicate degrading *S. aureus* (e.g. Fig. 1 Ai, blue arrow).

Notably, TEM revealed that bacteria-containing vesicles occasionally localized perinuclearly or closely associated with nuclear structures of SaOS‑2 (EDCC 5055 Fig. 1 Bi, Biii, EDCC 5464 Fig. 1 Bii, Biv), A549 cells, and, albeit less frequently, in U937 cells (Fig. S3). Given the images’ two-dimensional nature, the observed structures could merely represent a cross section of invaginations into the nuclear membrane, as frequently observed in other TEM images. Therefore, SBF-SEM confirmed juxtanuclear positioning in SaOS-2 across several sections, and, in a rare event, *S. aureus* was observed adjacent to nuclear structures without a clearly resolvable surrounding vacuolar membrane (Fig. 1 D-F). Importantly, the intact surrounding nuclear envelope could not be confirmed in these sections. Three-dimensional modelling of the nuclear surface using SIM-imaging also demonstrated strong colocalization with *S. aureus* based on both CFSE and DNA staining, and underscores the presence of large invaginations of the nuclear membrane for EDCC 5055 (Fig. 1G) and EDCC 5464 (Fig. 1H), at distinct relative frequencies (Fig. 1I). Isolate-dependent LAMP-1 proximity (Fig. 1J) in A549 (Fig. S3C-F) and SaOS-2 cells (Fig. S3G-J) indicated that a substantial fraction of bacteria-containing vesicles are consistent with lysosomal compartments, as reported for various strains previously [6, 10].

The detection of bacteria in nuclear-fractionation preparations provided additional, but, due to the possibility of co-sedimentation, technically non-definitive evidence of close nuclear association. EDCC 5055 and 5464 were enriched in both the cytosolic and nuclear fractions of each of the three cell lines (Fig. 1K), and bacteria remained viable in both fractions (data not shown).

### *S. aureus* infection induces a conserved transcriptional stress signature across isolates alongside a distinct, isolate-specific response in U937 cells

**Fig. 2:**
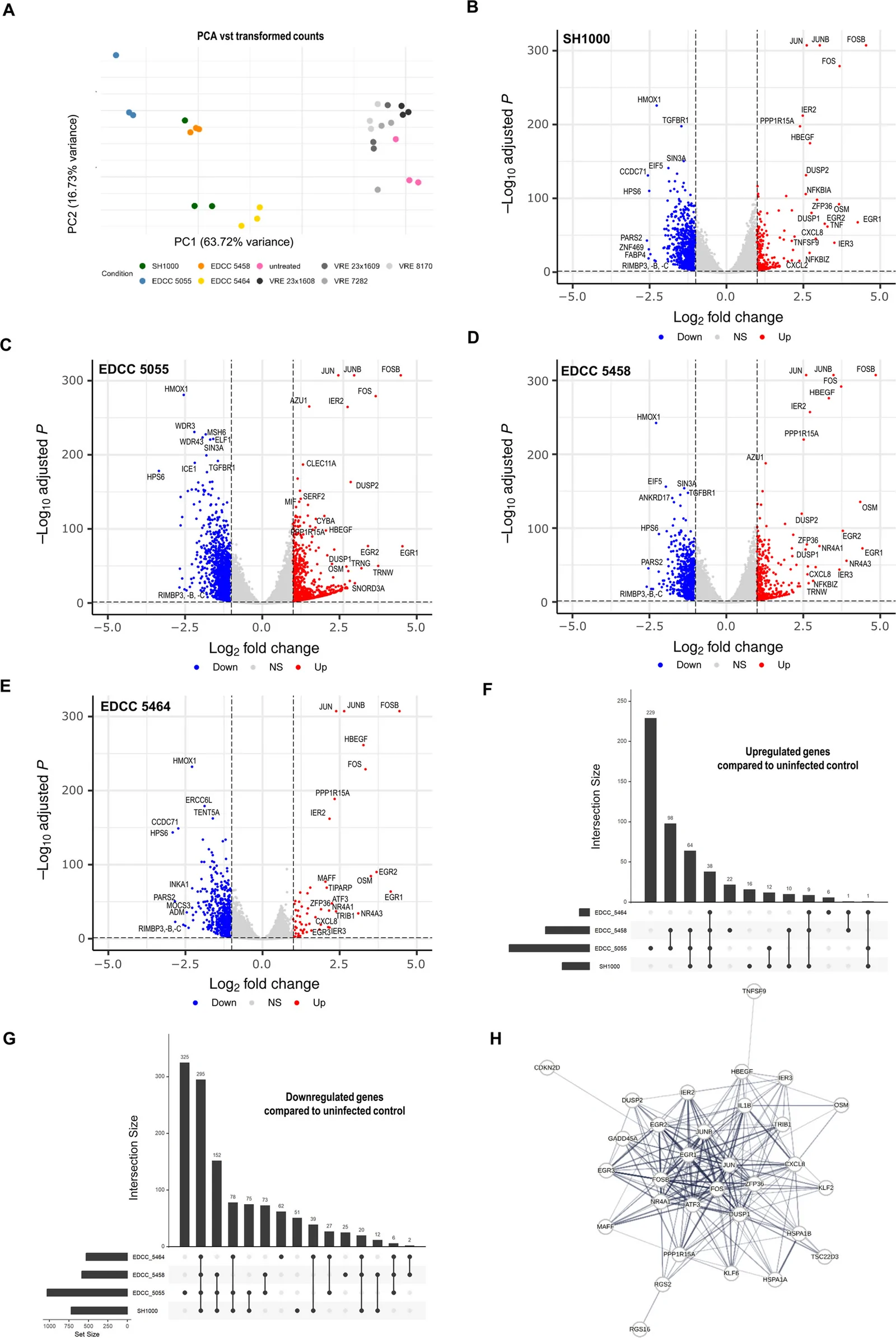
U937 cells infected with *S. aureus* reveal both shared and isolate-specific differentially regulated gene signatures. U937 cells were infected in triplicates with different *S. aureus* (4 hpi) using a gentamicin protection assay at MOI 30 or, for comparison, with *E. faecium* (6 hpi) isolates. The host’s transcriptional response was analyzed using Illumina mRNA sequencing. **A** Principal component analysis (PCA) of variance-stabilizing transformation (vst)-transformed data using the top 1000 most variable genes shows close clustering of VREfm-infected, but scattered clustering for *S. aureus*-infected cells compared to untreated controls. Further analyses are shown *for S. aureus* only. Volcano plots of cells infected with *S. aureus* isolates **B** SH1000, **C** EDCC 5055, **D** EDCC 5458, and **E** EDCC 5464 compared to uninfected control cells delineate overlapping and unique transcriptional regulation (padj < 0.05, |log2FC|≥ 1), supported by UpSet plots depicting overlap of significantly, at least two-fold **F** upregulated and **G** downregulated genes across conditions. **H** Full STRING network of shared upregulated, protein-coding genes (padj < 0.05, |Log2FC|≥ 1) across all *S. aureus* isolates (edges indicate confidence, medium confidence 0.400); disconnected nodes are not shown

Circulating and tissue-resident monocytes and macrophages are among the first line of defense countering invasive *S. aureus*. To characterize early host responses, we performed mRNA sequencing of U937 cells at 4 hpi (MOI 30)*.* Principal component analysis (Fig. 2A) revealed pathogen-specific host responses, with isolate-dependent clustering upon *S. aureus* infection, whereas the gram-positive control *E. faecium* induced comparatively uniform responses across isolates. Differentially expressed gene (DEG) analysis comparing *S. aureus* infection with the uninfected control (Fig. 2B-E) confirmed a divergent but strong transcriptional response, with a subset of highly regulated genes conserved across isolates (for interactive volcano plots, see Supplementary Files S4-S9). To identify functionally relevant expression patterns, subsequent analyses were restricted to significant protein-coding DEGs (padj < 0.05, |log2FC|≥ 1) induced by *S. aureus*.

The magnitude and composition of upregulated (Fig. 2F) and downregulated (Fig. 2G) genes varied strongly across isolates, with EDCC 5055 inducing the strongest and EDCC 5464 the weakest response. A shared core of 295 and 38 genes was down- and upregulated by all four isolates, respectively. This upregulated gene set (Fig. 2H) was among the top regulated and is consistent with an isolate-independent stress response, which transcriptionally regulates signal transduction, cell survival, and proliferation (e.g. *FOS*, *FOSB*, *JUN*, *JUNB*, *EGR1/2*, *IER2/3, HSPA1A/B*, *ZFP36*), as well as inflammation (*CXCL8*, *IL1B*).

**Fig. 3:**
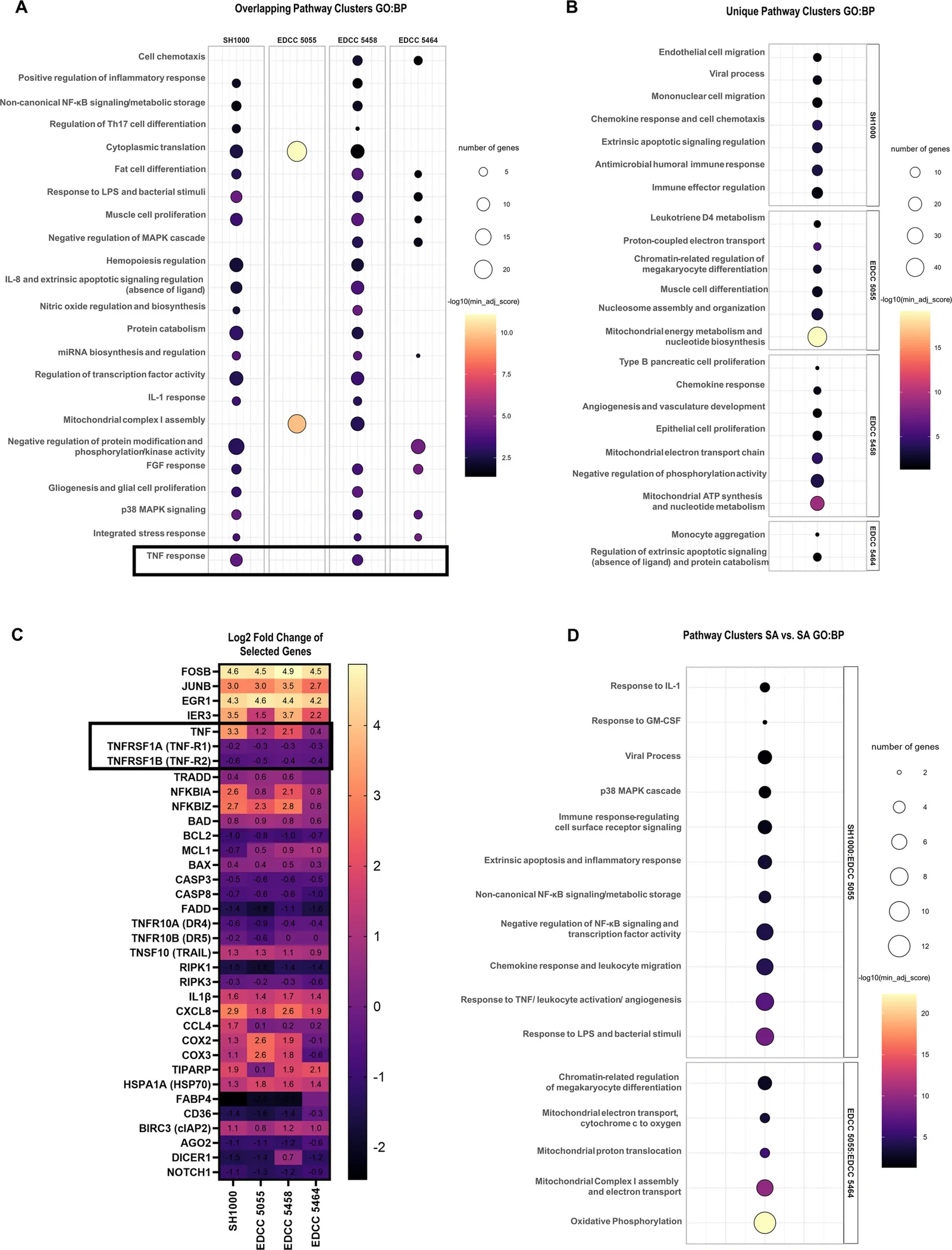
Intracellular *S. aureus* infection of U937 cells induces distinct upregulated pathway clusters dependent on each isolate*.* Significantly (padj ≤ 0.05), at least two-fold upregulated protein-coding genes were analyzed for enriched pathways (Gene Ontology Biological Processes) using g:Profiler ordered query. Based on Jaccard similarity of gene sets, hierarchical clustering (average linkage) with fixed cut height of 0.5 was performed. The adjusted enrichment score of a driver pathway (minimal padj) is shown representatively for each cluster (-log10(min_adj_score)). Bubble size indicates gene set size, for **A** overlapping and **B** unique pathway clusters of *S. aureus* isolates compared to untreated controls. **C** Log2 fold changes of 20 selected genes of interest against untreated control. **D** overlapping and unique clusters directly comparing responses of infected U937 cells (SH1000 vs. EDCC5055 and EDCC 5055 vs. EDCC 5464)

To functionally analyze shared (Fig. 3A) and unique (Fig. 3B) modulated pathways in infected versus uninfected control cells, we performed GO:BP pathway enrichment analysis and summarized the results via Jaccard hierarchical clustering (*h* = *0.5*) of significant gene sets. Directly comparing expression between cells infected with different isolates underscored distinct host response patterns (Fig. 3C, for SH1000 vs. EDCC 5055 and EDCC 5055 vs. 5464). Shared clusters still markedly varied in their respective enrichment scores and gene compositions across isolates.

Although EDCC 5055 triggered the strongest overall transcriptional reprogramming, the resulting response was functionally distinct and less enriched for canonical immune signaling compared to the other isolates. Instead, upregulated pathways were primarily linked to mitochondrial respiration and energy metabolism (e.g. *COX1, -*2, −3, *CYTB*, *MT-ND1, −2* or *ATP*6, −8), which were partly unique to this isolate (*MT-ND1*, *COX1*), alongside increased cytoplasmic translation (ribosomal proteins of the 40S and 60S subunits, e.g. *RPL*2*4*, *−26*, −31, *RPLP1*, or *RPS12*, *−14*, *−15*), and chromatin-organization associated pathways (e.g. *H4C1*,* −5–11*, *−12*, *−14*, *−15* or *H1-2, H1-4*). Inflammation-associated enrichment was minimal for EDCC 5055 and largely restricted to the single leukotriene D4 metabolism cluster (e.g. *DPEP2*, *GGT5*).

In contrast, the isolates EDCC 5458 and particularly SH1000 induced broader immune- and inflammation-related pathway activation that included genes related to chemokines and cytokines (*CCL20, CCL4, CXCL2, CXCL8, IL1B, TNF*), NF-κB regulators (*NFKBIA, NFKBIZ*), and to activation markers such as *CD69* (SH1000). These responses extended to pathways known to regulate apoptosis and cell survival (e.g. NF-κB–induced *BIRC3*, CIAP2, *TNF*, *NFKBIA*, *NFKBIZ*). TNF induction was particularly prominent among these isolates (log2FC 3.3 for SH1000, 2.1 for EDCC 5458), highlighting strong engagement of TNF-associated regulatory networks by these isolates that was absent for EDCC 5055 or 5464. In line with this, *TNFAIP3* (A20), encoding a key negative regulator of TNF signaling, was uniquely upregulated upon SH1000 infection, while it was downregulated in EDCC 5055-infected cells.

Consistent with these patterns, enrichment of immune- and apoptosis-related pathway clusters was observed for SH1000, EDCC 5458, and to a lesser extent EDCC 5464, driven by genes such as *CXCL8*, *IL1B*, *HSPA1A/B* (encoding HSP70), *TIPARP*, or *PP1R15A* (related to growth arrest and autophagy, lysosomal biogenesis). The regulation of MAPK signaling (e.g. *DUSP1*, *RGS2*, *GADD45A/B*) differed between isolates, with SH1000 showing positive regulation, EDCC 5464 predominantly negative regulation, and EDCC 5458 affecting both. EDCC 5055 did not show enrichment of these pathways despite modulation of individual genes, reinforcing its distinct transcriptional profile.

RIPK1, but not RIPK3, was significantly downregulated by all isolates. Several other key regulators of controlled cell death were significantly modulated by some isolates compared with uninfected controls, but they were below or near the cut off. These included anti-apoptotic genes, such as *BAD*, *BCL2*, or *MCL1*. Unlike *BAX*, many proapoptotic genes were downregulated, including caspase-3 and −8, aligning with moderate (over-cut-off) downregulation of *FADD*, or attenuated death receptor expression, e.g. *TNFRSF1A* (TNF-R1), *TNFR10A* (DR4) or *TNFR10B* (DR5; only for SH1000 and EDCC 5055). *TRADD* (not for EDCC 5464) and *TNSF10* (TRAIL) were moderately upregulated for all isolates below or near the cut-off. Neither *CD95/Fas*, nor *FasL* were transcriptionally regulated by any of the isolates. Selected genes of interest that were significantly regulated for at least one isolate and their log2FC values relative to the uninfected control are shown in Fig. 3C.

Direct comparisons of isolate-induced responses further emphasized these functional differences, as shown for SH1000 vs. EDCC 5055 and EDCC 5055 vs. EDCC 5464 (Fig. 3D). All pathway clusters upregulated by SH1000 compared to EDCC 5055 were related to immune responses, inflammation, and programmed cell death regulation. Compared to those of the low-response isolate EDCC 5464, pathway clusters upregulated by the high-response isolate EDCC 5055 were, except for chromatin-related regulation, entirely related to mitochondrial respiration.

Notably, the chronic infection isolate EDCC 5464 failed to induce substantial transcriptional regulation beyond the observed core response. For instance, in addition to the lack of inflammation and apoptosis regulation, genes involved in fatty acid metabolism and transport (*FABP4*, *CD36*) or RNA stability and processing (*AGO2*, *DICER1*, *DHX36*) were downregulated by all isolates except EDCC 5464, consistent with metabolic and posttranscriptional changes (Supplementary Fig. S10). Additional downregulated pathway clusters included Notch activation (e.g., *NOTCH1*, *MAML1-3*) (SH1000 and EDCC 5458), while EDCC 5055 suppressed pathways related to p53-mediated (e.g., *MYC*) or TGF-β (e.g., *TGFBR1*) signaling, epigenetic regulation of gene expression (e.g. *ALKBH-1*/*−4*), small GTPase signaling (e.g. *ABL2*), or ribosome biogenesis (e.g. *DDX28)*, among others. The full lists of DESeq2 data, overall significant, unique and shared DEGs as well as enriched pathways, clusters, and included genes are available in Supplementary File S11.

Across cell types, these isolate-specific transcriptional programs were partly mirrored by distinct cytokine release patterns (Supplementary Fig. S12), including prominent CXCL8 (IL-8), but variable TNF release that was most pronounced in SH1000-infected U937 cells, matching the inflammatory signature of this isolate. TNF release was not detected in A549 and SaOS-2 cells. Maintained or even enhanced responses to nonviable bacteria underscored that both the bacterial background and the host context shape downstream inflammatory signaling and cell fate decisions.

Together, these data demonstrate that while a conserved stress response was induced across all *S. aureus* isolates, isolate-specific transcriptional programs strongly diverged. These differences were most pronounced in pathways related to mitochondrial metabolism (EDCC 5055) versus TNF-associated inflammation and cell death regulation (SH1000, EDCC 5458), whereas EDCC 5464 showed minimal host activation beyond the shared core response.

### Intracellular *S. aureus* induces heterogeneous cell-death-associated responses in U937 monocytes

Intracellular *S. aureus* is known to cause highly heterogeneous cell death depending on both the strain and the infected host cell type [20–22]. Thus, we next investigated isolate-dependent host cell fates by analyzing infection-induced cell death in U937 cells compared with A549 and SaOS-2 cells.

**Fig. 4:**
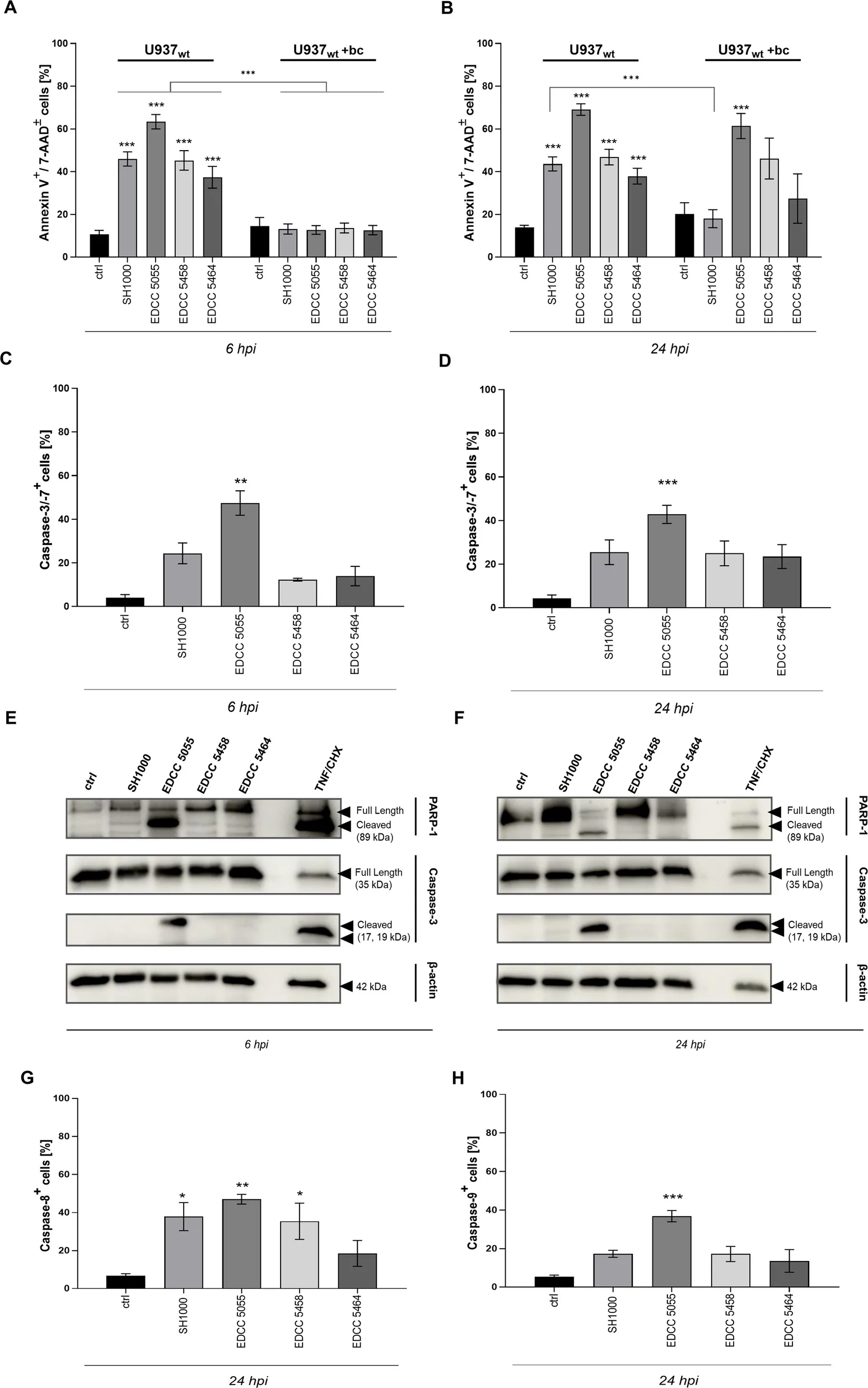
Intracellular *S. aureus* induces cell death of U937 cells to an isolate-dependent extent. Cells were infected with *S. aureus* isolates at MOI 30 using a gentamicin protection assay or direct contact being prevented using Boyden chamber inserts (+ bc). **A**, **B** At 6 hpi and 24 hpi AnnexinV^+^/7-AAD^±^ cells, **C**, **D** Caspase-3/−7^+^ at 6 hpi and 24 hpi. Protein expression (20 µg) of the full-length and cleaved form of PARP-1 and caspase-3 was confirmed via Western Blot at **E** 6 hpi and **F** 24 hpi, including β-actin as loading control. **G** Caspase-8^+^ and **H** Caspase-9.^+^ cells at 24 hpi were quantified using flow cytometry. Data show mean ± SEM of n ≥ 3 independent experiments. Cells were sensitized with cycloheximide (CHX) (0.5 µM) 30 min prior to treatment with TNF (100 ng/mL) as a positive control. Statistical analysis was performed using Two-way ANOVA with Tukey’s Multiple Comparisons between all conditions. Significance is shown for each *S. aureus* isolate versus uninfected control as well as for the same isolate between groups (with and without bc; indicated by lines); not shown for isolate versus isolate within the same group. *p < 0.05; **p < 0.01; ***p < 0.001

In U937 cells, Annexin V/7-AAD staining revealed host cell death signaling by al. *S. aureus* isolates as early as 6 hpi that was most pronounced upon challenge with EDCC 5055 (Fig. 4A, left). Notably, preventing direct bacteria‒host cell contact (bc: boyden chamber), cell death was abrogated at 6 hpi, emphasizing the need for contact or uptake (Fig. 4A, right). Without bc, the degree of isolate-specific host cell death did not substantially increase from 6 to 24 hpi (Fig. 4B, left). However, with bc, cell death induction was significantly restored for EDCC 5055 at 24 hpi, and tended to re-occur for EDCC 5458 and 5464 (Fig. 4B, right). Significant activity of the proapoptotic caspases-3 or −7 was observed only for EDCC 5055 at 6 hpi (Fig. 4C, E) and then barely further increased until 24 hpi for all isolates (Fig. 4D, F), consistent with early engagement of apoptosis-associated signaling, particularly during EDCC 5055 infection. Notably, both actin and full-length caspase-3 were only moderately reduced upon a strong apoptotic stimulus (TNF/CHX), suggesting that infection did not affect β-actin stability (Fig. 4E, F). Moderate differences in β-actin density may be due to bacteria-containing samples containing proteins from both species that resulted in lower actin signals due to reduced host protein proportions. In such cases, signals such as cleaved PARP-1 or cleaved caspase-3 would be underestimated, however, the effects aligned with those of flow cytometry.

Despite minimal caspase-3/−7 effector activity, all isolates except EDCC 5464 elicited significant activation of the initiator caspase-8 at 24 hpi (Fig. 4G), consistent with the engagement of extrinsic apoptosis-related signaling. Significant caspase-9 activity was observed exclusively in cells exposed to EDCC 5055 (Fig. 4H), compatible with additional mitochondrial apoptosis-associated signaling. However, a proportion of the cells lacked activity of these proapoptotic caspases, but were Annexin V and/or 7-AAD–positive, and pan-caspase inhibition using zVAD.fmk did not prevent cell death (data not shown). Of note, both necroptosis and pyroptosis are reported to permit 7-AAD uptake and Annexin V is shown to also marks pyroptotic cells [66].

### TNF-R1 signaling is critical for the timing and extent of isolate-specific host cell death induced by intracellular *S. aureus* in U937 monocytes, but not in A549 or SaOS-2 cells

**Fig. 5:**
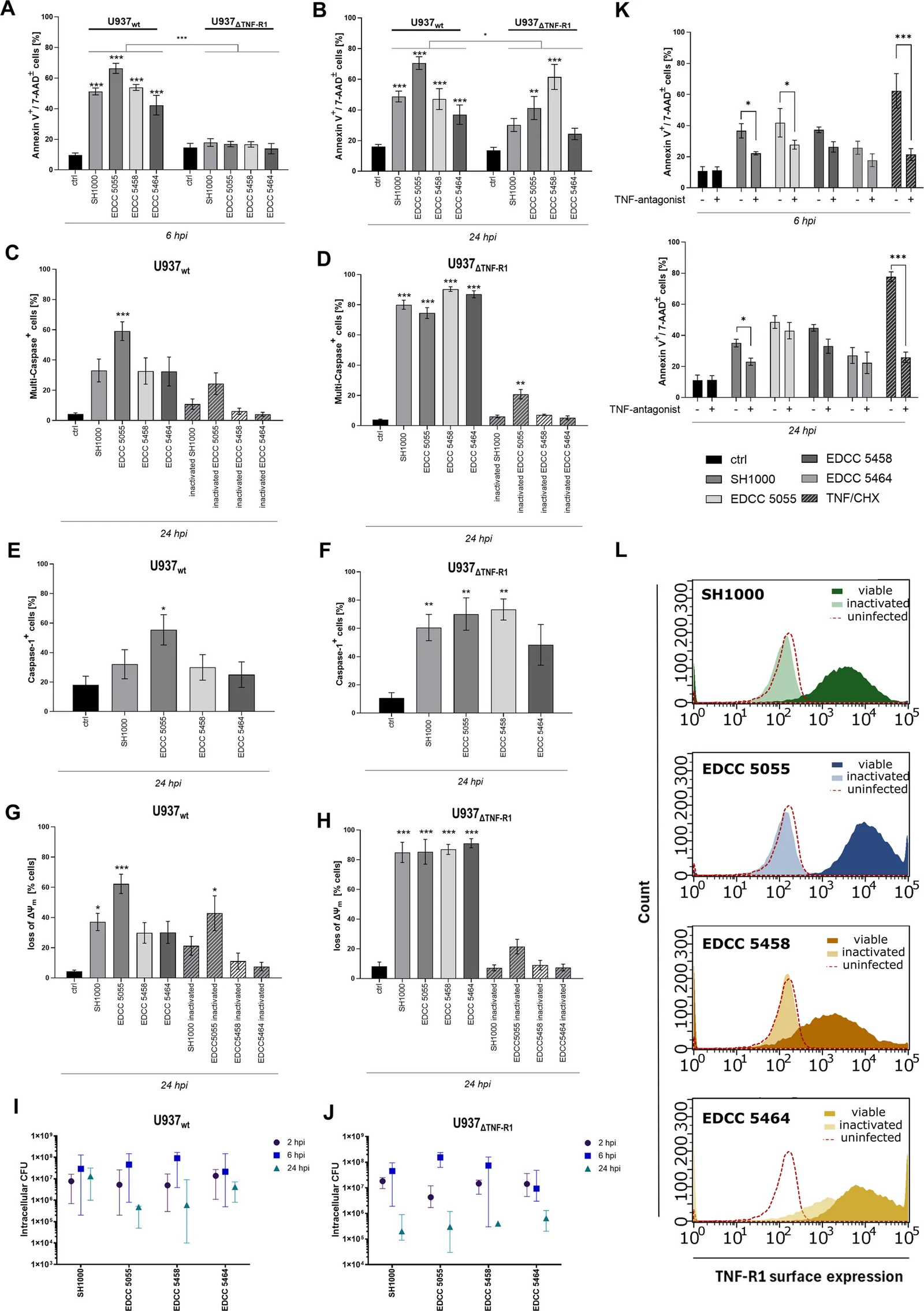
TNF-R1 signaling influences the timing and magnitude of isolate-specific U937 cell death during intracellular *S. aureus* infection. U937 wildtype and U937_ΔTNF-R1_ cells were infected with viable or heat-inactivated *S. aureus* isolates at MOI 30 using a gentamicin protection assay. **A** At 6 hpi and **B** 24 hpi, Annexin V^+^/7-AAD^±^ cells (Dead Cells) were analyzed using flow cytometry. At 24 hpi, Multi-Caspase^+^, Caspase-1^+^, and depolarized (loss of mitochondrial transmembrane potential, ΔΨm) cells were quantified by flow cytometry in **C**, **E**, **G** U937_wt_ and **D**, **F**, **H** U937_ΔTNF-R1_ cells, respectively. **I** U937_wt_ and **J** U937_ΔTNF-R1_ cells were lysed at the indicated time-points, and intracellular CFU were quantified per lysate (initial concentration: 1 × 10^6^ cells). **K** Annexin V^+^/7-AAD.^±^ cells (Dead Cells) with or without the TNF-antagonist Fab’ at 6 and 24 hpi. **L** TNF-R1 surface expression of infected and uninfected cells at 2 hpi. Data show **A**-**H**, **K** mean ± SEM of n ≥ 3 independent experiments **I**, **J** median ± 95% CI on log-scale, zero-values were excluded, **L** representative histograms of n ≥ 3 experiments. Statistical analysis was performed comparing means between all conditions. Significance is shown for each *S. aureus* isolate versus uninfected control as well as for the same isolate between groups (*wt* vs. *ΔTNF-R1*; ± TNF-antagonist; indicated by lines); not shown for isolate versus isolate within the same group. *p < 0.05; **p < 0.01; ***p < 0.00

While the TNF–TNF-R1 system is known to be essential for regulating the immune surveillance of many pathogens, the underlying mechanisms are not fully understood. For example, patients receiving anti-TNF treatment are frequently more susceptible towards infections like *S. aureus* [67–70]. In this study, we observed isolate-specific TNF transcriptional regulation with activation of TNF-related proinflammatory and survival pathways in U937 cells and variable cytokine release, implying the TNF–TNF-R1 system to influence infection outcomes. Markedly, *S. aureus*-induced host cell death (Annexin V^+^/7-AAD^±^) was completely prevented in U937 cells lacking TNF-R1 (U937_ΔTNF-R1_) at 6 hpi (Fig. 5A), but was restored at 24 hpi; however, at significantly different levels compared with U937_wt_ cells for each isolate (Fig. 5B). Simultaneous multi-caspase profiling revealed isolate-specific activity that exceeded that caspase-3/−7, −8, or −9 alone at 24 hpi (Fig. 5C), while multi-caspase and caspase-3/−7 activity were comparable at 6 hpi (Supplementary Fig. S13). These observations suggest that, particularly at later stages of infection, cell death induction might involve additional pathways in both the presence and absence of TNF-R1.

Notably, excessive multiple-caspase activation was induced in U937_ΔTNF-R1_ cells at 24 hpi by all isolates, even EDCC 5464, negating previously observed isolate-specific differences (Fig. 5D). The extent of caspase-1 activity in U937_wt_ cells was comparable to that of multiple-caspases at 24 hpi (Fig. 5E), reflecting pathway activation compatible with inflammasome- and caspase-1–associated signaling during later stages of infection, although definitive cell death modalities like pyroptosis could not be established. Caspase-1 activation levels were again markedly amplified in U937_ΔTNF-R1_ cells (Fig. 5F).

Disruption of mitochondrial transmembrane potential (ΔΨm), which is linked to several cell death modalities, of U937 cells occurred in response to SH1000 and EDCC 5055 (Fig. 5G) and was potentiated in TNF-R1–deficient cells across all tested isolates (Fig. 5H). In both U937_wt_ and U937_ΔTNF-R1_ cells, several of these effects were restricted to viable *S. aureus* for all isolates except EDCC 5055.

The observed discrepancy between U937_wt_ and U937_ΔTNF-R1_ cells might be attributed to differential uptake efficacy. The quantification of intracellular CFUs per lysate revealed comparable uptake rates in both wild-type (Fig. 5I) and TNF-R1–deficient (Fig. 5J) U937 cells. The number of CFUs slightly increased from 2 to 6 hpi, hinting towards bacterial replication, followed by a variable decline from 6 to 24 hpi. This pattern is consistent with the observed cell death induction and might thus account for bacterial release rather than bacterial degradation. In support of this, we did not detect quantifiable differences between isolates e.g. in bacterial fluorescence intensity or spot counts by imaging flow cytometry analyses at 2 hpi (Fig. 1 Av-viii). Equally, flow cytometry of CFSE-labeled bacteria showed comparable uptake rates of viable and inactivated isolates (not shown).

Interestingly, the addition of TNF-antagonizing Fab’, intended to scavenge liberated TNF, did not affect cell fates at equivalent rates compared to TNF-R1 knockout. A partial rescue was observed at 6 hpi that decreased until 24 hpi except for SH1000 (Fig. 5K). On the other hand, infection with each viable isolate led to increased TNF-R1 surface expression in U937 cells at 2 hpi (Fig. 1L). Except EDCC 5464, receptor expression during infection with inactivated *S. aureus* was comparable to uninfected cells.

**Fig. 6:**
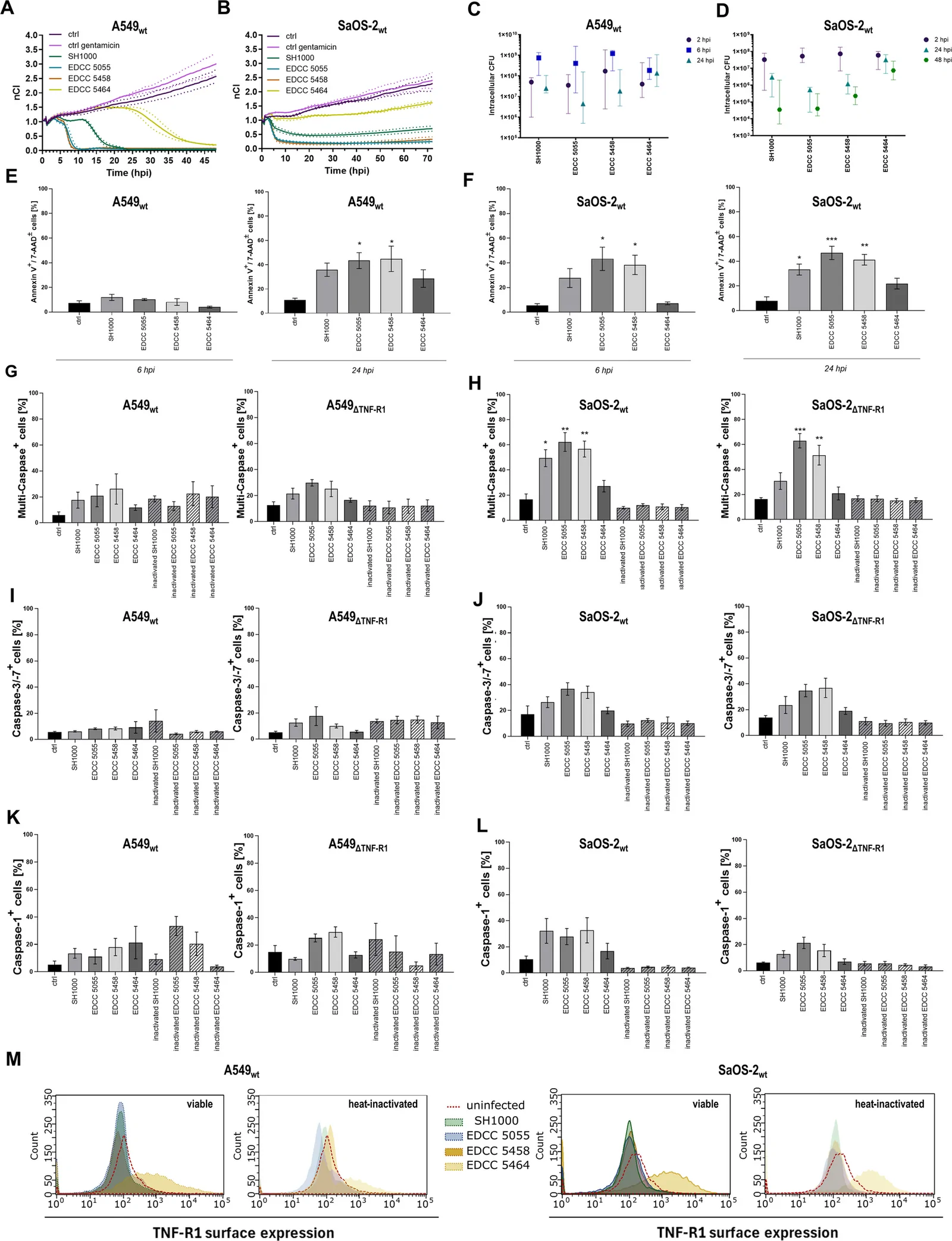
Differential cell death patterns of A549 and SaOS-2 during intracellular *S. aureus* infection that are only moderately modulated by TNF-R1 signaling. A549_wt_, A549_ΔTNF-R1_, SaOS-2_wt_, and SaOS-2_ΔTNF-R1_ cells were infected with viable or heat-inactivated *S. aureus* isolates at MOI 30 using a gentamicin protection assay. Cell death kinetics of infected, untreated, and gentamicin-treated control **A** A549_wt_ and **B** SaOS-2_wt_ cells were normalized to the time of infection using Real-time cell analysis. Detachment of cells used as surrogated for cell death is depicted as decreasing normalized Cell Index (nCI); one representative experiment is shown as mean ± SD (dotted line). At the indicated time-points, **C** A549_wt_ and **D** SaOS-2_wt_ cells were lysed, and intracellular CFU were quantified per lysate (initial concentration: 5 × 10^5^ cells). Data show Median ± 95% CI on log-scale, zero-values were excluded. **E**, **F** Cell death was confirmed by AnnexinV/7-AAD staining at 6 hpi and 24 hpi. Caspase activation was assessed at 24 hpi, for **G**, **H** Multi-Caspase^+^, **I**, **J** Caspase-3/−7^+^, and **K**, **L** Caspase-1^+^ cells by flow cytometry in **G**, **I**, **K** A549_wt_ (left) and A549_ΔTNF-R1_ (right) and **H**, **J**, **L** SaOS-2_wt_ and (left) SaOS-2_ΔTNF-R1_ (right) cells, respectively. Data show mean ± SEM of *n* ≥ 3 independent experiments. **M** Representative experiment of TNF-R1 surface expression in infected and uninfected A549 (left) and SaOS-2 (right) cells at 2 hpi. Statistical analysis was performed using One-way ANOVA with Tukey’s or Kruskal–Wallis Multiple Comparisons. *p < 0.05; **p < 0.01; ***p < 0.001 vs. uninfected controls

To delineate *S. aureus*-induced cell fates of the used nonphagocytic cell lines, RTCA demonstrated isolate-dependent cell detachment used as a surrogate for cell death of A549 (Fig. 6A) and SaOS-2 (Fig. 6B) at distinct kinetics and extents. EDCC 5055 and EDCC 5458 induced rapid detachment in both A549 and SaOS-2 starting at approximately 5 hpi. SH1000 simultaneously initiated cell detachment, which then proceeded more gradually. In contrast, EDCC 5464-induced detachment was delayed by approximately 24 h and proceeded slowly within 48 hpi in A549 cells, and was absent in SaOS-2 cells up to 72 hpi while growth rates stagnated. Correspondingly, intracellular CFUs of all isolates except EDCC 5464 declined in both A549 (Fig. 6C) and SaOS-2 cells (Fig. 6D). Annexin V/7-AAD analysis confirmed these host death rates for A549 (Fig. 6E) and SaOS-2 cells (Fig. 6F) at 6 and 24 hpi.

Next, we assessed caspase activation and the impact of TNF-R1 in these cell lines. Compared to U937 monocytes, activity of multiple caspases (Fig. 6G, left), caspase-1 (Fig, 6 K, left) and caspase-3/−7 (Fig. 6I, left) was less pronounced or absent in A549_wt_ cells at 24 hpi. The knock-down of TNF-R1 only slightly reinforced these activities (Fig. 6G, I, K, right). In both A549_wt_ and, for caspase-1, in A549_ΔTNF-R1_, heat-inactivated *S. aureus* also resulted in effector-dependent stimulation, partially exceeding that of their viable counterparts.

In SaOS-2 cells, significant activation of multiple caspases (Fig. 6H) was induced by viable SH1000, EDCC 5055, and EDCC 5458 in wild-type cells at 24 hpi (Fig. 6H, left), whereas this response was lost for SH1000 in TNF-R1 deficient cells (Fig. 6H, right). In contrast to U937 cells, caspase-3/−7 (Fig. 6J) and −1 (Fig. 6L) were activated with reduced isolate-specific differences in either cell type, and caspase-1 activation was slightly attenuated in SaOS-2_ΔTNF-R1_ compared to SaOS-2_wt_ cells. TNF-R1–deficiency retained isolate-specific effects in both A549 and SaOS-2 cells, albeit being less pronounced in the first place. Interestingly, TNF-R1 surface expression was increased by viable and heat-inactivated EDCC 5464, but not the other isolates (Fig. 6M; individual histograms see Fig. S13D).

Taken together, these findings emphasize cell line- and isolate-dependent host cell fates during intracellular *S. aureus* infection. Cell death involved heterogeneous activation of caspases consistent with apoptotic pathway engagement particularly in U937 and SaOS-2 cells, but likely involved other modalities, whose definitive identification requires further validation. The effects observed upon TNF-R1 knock-down indicate a biphasic role of this receptor in regulating cell death of U937 monocytes, which was associated with delayed early cell death signaling followed by a later increase in broad effector activation and loss of cell viability with reduced isolate-specific differences. However, a comparable impact was not observable in A549 and SaOS-2 cells. These effects required viable bacteria (except EDCC 5055) and TNF-R1 surface expression was heterogeneously affected across cell types, underscoring the highly specific *S. aureus*–host interactions to influence host cell death versus survival.

## Discussion

Intracellular infection of various professional and nonprofessional phagocytic host cell types is now a well-studied feature of *S. aureus*. However, many studies focus on aspects of host cell modulation in single cell lines induced by an individual isolate, neglecting the interplay of specific isolate- and tissue type-dependent properties to shape the cellular outcome [6, 9, 71]. In this study, we demonstrate that intracellular *S. aureus* elicited a conserved early stress response across four isolates yet triggered highly distinct transcriptional as well as isolate- and cell type-specific cell death programs. In U937 monocytes, TNF-R1 appeared to influence the timing and magnitude of cell-death–associated responses, whereas its contribution to infection outcomes of A549 and SaOS-2 cells was much less pronounced. An ancillary observation indicated frequent peri- and juxtanuclear association of *S. aureus* across cell types, although the functional relevance of this positioning remains unknown.

### Isolate-specific transcriptional programs of U937 monocytes in response to intracellular* S. aureus*

Transcriptomic profiling of U937 monocytes during intracellular *S. aureus* infection revealed a robust, conserved stress response across isolates at 4 hpi, which included the upregulation of immediate-early and inflammatory genes (e.g. *FOS*, *JUN*, *OSM*, *CXCL8*, *IL1B*) [72]. In parallel, the isolates elicited highly divergent transcriptional signatures: SH1000, EDCC 5055, and EDCC 5458 induced hundreds of DEGs, whereas EDCC 5464 infection was accompanied by a comparatively muted response. Because intracellular CFUs were comparable across isolates until the onset of host cell death at approximately 6 hpi, and bacterial loads remained stable for EDCC 5464 even up to 24 hpi, these differences are more likely to reflect genuine isolate-specific host–pathogen interactions than variation in bacterial uptake.

Beyond the shared core response, each *S. aureus* isolate triggered unique pathway activation. EDCC 5055 most prominently upregulated mitochondrial energy metabolism pathway clusters, consistent with a cellular response involving mitochondrial processes and stress-associated remodeling, as previously described for *S. aureus* [73, 74]. Particularly SH1000 and also EDCC 5458 robustly provoked e.g. TNF, NF-κB, or MAPK-related inflammatory and apoptosis-related programs. Correspondingly, both isolates triggered moderate TNF release in U937 cells at 4 hpi, but not in A549 and SaOS-2 cells. This finding is consistent with reports showing that monocytes and macrophages are the main source of soluble TNF [75], involving maximum mRNA production at 3 h and maximum TNF release at 5–6 h post stimulation [76, 77]. Supposedly, the unique up- and downregulation of related genes such as *TNFAIP3* by SH1000 and EDCC 5055, respectively, are consistent with finely-controlled TNF expression and affect apoptotic signaling in infected cells [78, 79]. All isolates except EDCC 5464 downregulated *FABP4* and *CD36* transcription, linked to fatty acid metabolism. The latter has also been implicated as a phagocytic receptor for *S. aureus,* with CD36-deficiency impairing TNF production and bacterial clearance [80].

Notably, inflammatory pathway enrichment was largely absent not only for the chronic isolate EDCC 5464, but also for the highly cytotoxic EDCC 5055. One possibility is that these patterns reflect isolate-specific strategies to modulate inflammatory and immune signaling [78, 81–83]; however, immune evasion was not directly tested in this study. Genes related to leukotriene metabolism such as *DPEP2* were upregulated by EDCC 5055, and this gene was previously reported to act as a cell-type specific regulator for macrophage-mediated inflammation by repressing NF-κB and MAPK activity as well as TNF, IL-6, or MCP-1 release [84, 85]. Appropriate functional interpretation of these observations, however, will require independent validation of key genes and pathways.

Besides the downregulation of a signaling cluster associated with NLS-bearing nuclear import, each isolate suppressed genes governing RNA stability and catabolism regulation compared with uninfected U937 cells. Dysregulated RNA stability may contribute to the host transcriptional differences observed during infection [86], although its consequences for bactericidal activity were not assessed. EDCC 5055 further suppressed p53-linked signaling and epigenetic regulators while upregulating chromatin-organization genes, an observation that is consistent with epigenetic dysregulation previously reported for intracellular *S. aureus* infection [11, 87]. Accordingly, whereas *S. aureus*-induced neutrophil lysis has been shown to require contact-independent factors such gene transcription and protein synthesis [65], key cellular pathways will likely also rely on transcription-independent regulation [17]. Given the observed differential regulation of phosphorylation and protein-modification pathways in the U937 transcriptome, targeted organelle proteomic analyses, including post-translational modifications, and profiling of noncoding RNAs such as miRNAs may help resolve key signaling mechanisms. Various *S. aureus*-induced transcriptional changes in nonprofessional phagocytes, such as osteoblasts [11] and endothelial cells [7, 78], are described to both intersect and diverge from the U937 responses observed in the current study. Cell line-specific effects and comparisons with primary cells will be essential to delineate tissue-dependent host reactions.

### Differential host cell death responses across isolates and cell types

The manipulation of timing and mode of host cell death is a key strategy for bacterial escape and dissemination. Intracellular fates of individual *S. aureus* isolates are reported to show common traits across nonphagocytic cells but differ from those in macrophages, underscoring the relevance of host cell context in bacterial persistence [9]. Consistent with previous reports [6, 9, 88–90], our data illustrate cell line- and isolate-specific death rates, kinetics, and engaged signaling pathways.

Among the cell lines tested in this study, susceptibility to *S. aureus*-induced cell death was heterogenous, with rapid and pronounced host cell destruction of U937 monocytes, followed by SaOS-2 osteoblast-like cells, while A549 lung epithelial cells were less sensitive. The isolates EDCC 5055 and EDCC 5458 mounted the strongest, while SH1000 and EDCC 5464 elicited delayed or weaker responses across cell lines. However, A549 cells showed only marginal caspase activation despite clearly detectable detachment and Annexin V/7-AAD staining, indicating that caspase activation alone is insufficient to explain the observed loss of viability.

These observations are compatible with reports showing that pan-caspase inhibition does not necessarily prevent cell death and that loss of viability can occur despite concurrent caspase activation [91, 92]. Further, our flow cytometric analyses indicated the presence of distinct subpopulations; hence fluorescence-based sorting could help distinguish infected from noninfected cells to determine whether caspase-negative survivors resist, avoid or carry persistent *S. aureus* infection [73]. In line with other studies, cytotoxicity was largely restricted to viable bacteria [93, 94], with moderate host cell damage induced only by heat-inactivated EDCC 5055.

Our findings suggest that particularly cell death of U937 monocytes progressed through different signaling stages between 6 and 24 hpi. Early cell death required direct contact or uptake, as shown by significant host cell rescue using bc-inserts, and involved isolate-dependent activation of caspases-3/−7, −8, and −9. Together with the observed changes in the mitochondrial transmembrane potential, these effects are consistent with the engagement of extrinsic and, for EDCC 5055, additional intrinsic apoptotic signaling. At 24 hpi, however, bc-inserts failed to still significantly prevent cell death for all isolates except SH1000, indicating that specific bacterial factors or other contact-independent effects may contribute to late-stage loss of host cell viability. Of note, as bacteria remained present under the bc-separated conditions, contact-independent factors such as nutrient availability might have contributed to this observation. Although the reduced uptake of some isolates, including SH1000, has been linked to altered cytotoxicity profiles [6, 95], other studies report no such association [6, 9, 10, 96]. Inconsistent plating yields caused by bacterial aggregation and nondividing phenotypes limit the interpretability of CFU quantification [7, 97], underscoring the need for parallel high-resolution microscopy. Nevertheless, differential uptake is unlikely to account for the observed isolate- and cell-type–specific outcomes in the current study.

In parallel, we observed a plateau of U937 cells staining positive for apoptosis-related caspases that coincided with isolate-dependent caspase-1 activity, compatible with inflammasome/caspase-1–associated signaling during later stages of infection. Gasdermin D or E cleavage at the protein level was not detected via Western blot (not shown), while enhanced IL-1β transcription was induced in U937 cells across isolates at 4 hpi. The release of this cytokine, however, was not observable in Luminex screenings at 1 or 4 hpi. For mature IL-1β release from cells, at least two specific signals must be activated, which include a transcriptional response leading to the production of pro-IL-1β and a second signal causing pro-IL-1β processing by caspase-1 [98]. Given the observed timing of caspase-1 activation, future analyses should assess IL-1β release at later time points and include additional markers such as inflammasome activation and HMGB1 to enable definitive assignment of the involved cell death phenotype, for example pyroptosis. MLKL oligomerization, used here as a marker of necroptosis-associated signaling, was not detected by Western blotting (not shown). Notably, many cell lines, including A549 cells, lack RIPK3 expression and are therefore incapable of necroptotic death [99].

These findings are in support of available studies linking mixed cell death modalities to intracellular *S. aureus* infection that can occur distinctly or concomitantly depending on the infection stage and host cell types [65, 66, 100]. For example, human neutrophils undergo apoptosis prior to (independently proceeding) lysis [65]. Conversely, macrophages and epithelial cells that survive initial α-hemolysin–driven inflammatory death exhibit apoptotic features only at later infection stages [101, 102]. Moreover, resource competition within the intracellular niche drives temporal shifts in bacterial and host metabolism, influencing bacterial replication, subcellular localization, and apoptosis of lung epithelial cells [73]. *S. aureus* cell wall degradation in macrophage phagosomes is shown to be functionally linked to the activation of the inflammasome and IL-1β, but not TNF-α secretion, which can be subverted by the pathogen through peptidoglycan modification [98]. On the other hand, whereas NLRP3-dependent monocyte necrosis can be induced by α-hemolysin or LukAB by extracellular *S. aureus*, LukAB-mediated necrosis is independent of ASC or NLRP3 by phagocytized *S. aureus* [101, 103], but shares common features with pyroptotic activation. Hence, experimental conditions (e.g. assay endpoint, model, strain choice and intracellular lifestyle) appear to strongly shape the molecular signaling underlying the observed outcomes, which can be highly heterogenous despite depending on shared effectors.

The low-response phenotype of EDCC 5464 observable throughout our analyses likely reflects frameshift mutations in *agrC* and *graS*, mobile-element insertions in β-hemolysin, and the absence of additional cytotoxicity-associated genes such as *lukED*, *splABC*, and *esxB* [37]. Proteomic profiling further showed a lack of PSM and splBDEF expression prior to infection, unlike the other isolates. Notably, esxA can inhibit apoptosis and is, together with esxB, required for bacterial release [104]; EDCC 5464 retains esxA but lacks esxB, features compatible with a persistent, noncytotoxic, nonescape phenotype. Phagolysosomal escape and subsequent cytosolic replication are often considered prerequisites for host cell death, especially in nonprofessional phagocytes [10, 105], though both processes can occur independently in a strain- and cell-type–specific manner [6, 65]. It requires subcellular manipulation, such as blocking lysosomal acidification or disrupting membranes, driven by agr/saeRS regulators and toxins including α-/β-hemolysin, PSMs, or PVL, whereas other studies highlight the impact of systems like graRS or sigB [3, 65, 93, 102, 106–108]. Their contributions vary with experimental conditions, and factors such as leukotoxins/PVL show strong species specificity, predominantly targeting human professional phagocytes [89, 103, 109]. Accordingly, ongoing work using CWT-YFP reporter cell lines [110] suggests reduced phagolysosomal escape of EDCC 5464 versus EDCC 5055 in SaOS-2 cells, which is correlated with host cell death (not shown). Thus, analysis of isolate-specific subcellular localization at different MOIs, specific compartmental markers such as LC3, LAMP-1/−2, acidification, or TNF-R1 co-internalization, and phagolysosomal escape across cell lines will help clarify the mechanisms underlying reduced cytotoxicity and *S. aureus* intracellular persistence.

Although the isolates share key virulence factors at the genomic and steady-state proteomic levels, the absence of secretome quantification during infection remains a major limitation of these analyses. Defining toxin expression across cell lines will help explain divergent host responses, as e.g. varying α-hemolysin levels can trigger distinct death pathways within the same cell type [91, 111]. Consistent with this, Strobel et al*.* reported low, context-dependent cytotoxicity of SH1000, with reduced α-hemolysin, other hemolysins, and PSMs [6], which may explain the isolate-specific lack of U937 killing when direct contact was prevented.

### The regulatory role of the TNF–TNF-R1 axis in the infection outcome of U937 monocytes

Remarkably, TNF-R1 deficiency restored U937 viability during early stages of infection, yet promoted broad host cell death induction with pronounced multi-caspase activation and mitochondrial depolarization at later stages across *S. aureus* isolates; a phenotype that was not equivalently induced by Fab’-mediated TNF scavenging and was far less pronounced in A549 and SaOS-2 cells. In U937 transcriptomic analyses, TNF-related signaling was either strongly engaged or widely bypassed depending on the isolate. This discrepancy suggests that distinct receptor- and ligand-dependent mechanisms contribute to the balance between host cell death and survival during intracellular *S. aureus* infection, although their mechanistic roles remain to be resolved.

TNF activates pro-inflammatory/pro-survival signaling cascades via TNF-R1-bound *complex I* formation, which culminate in NF-κB and MAPK activation. Under conditions where these pathways are insufficient or blocked, TNF-R1 signaling can instead promote cell death [23]. In addition, endocytosed TNF-R1 has been reported to contribute to bacterial degradation [112–115]. We found that all tested isolates increased TNF-R1 surface abundance early after infection in U937 cells, whereas only EDCC 5464 induced a comparable increase in the nonphagocytic cell lines. The functional consequences of this observation and its relationship to TNF-R1–dependent regulation of host cell death remain unresolved. Several mechanisms could account for the altered receptor abundance and should be investigated in future studies, including enhanced transport from the Golgi to the plasma membrane, reduced receptor internalization or shedding, and other infection-induced regulatory processes such as ligand-independent receptor activation with downstream effects on cell-death–associated signaling [116, 117].

These findings may also have implications for human infection and therapy. TNF, TNF-R1, and downstream effectors are essential for clearing intracellular pathogens, and impaired TNF activity is associated with infection susceptibility [118, 119]. In line with this, exogenous TNF was shown to prevent *S. aureus* infection in guinea pigs [120]. More recently, a case–control study reported significantly increased risk of blood stream infections, especially caused by *S. aureus* or *E. faecium*, in patients receiving anti-TNF therapy [119]. Given that infections during TNF inhibition are often discussed in the context of ‘classical’ intracellular pathogens such as *Mycobacterium tuberculosis* and *Listeria monocytogenes* [121], acknowledging the facultative intracellular survival of common pathogens like *S. aureus* is highly relevant in the context of TNF/TNF-R1 signaling. At the same time, TNF-directed interventions have shown benefit e.g. in nonbacterial osteomyelitis by attenuating bone loss [122] or *S. aureus*-mediated arthritis, where ADAM17 inhibition reduces TNF release [123, 124], underscoring that this pathway can be therapeutically relevant in both directions depending on context.

Because of side effects associated with anti-TNF therapy, selective TNF-R1 inhibition has been proposed as an alternative target [125, 126]. Our findings raise the possibility that TNF-R1–targeted interventions could alter the fate of infected phagocytes and thereby affect bacterial containment that may lead to unintended consequences via enhanced cytotoxicity and inflammation [4, 16–19]. This is consistent with the idea that *S. aureus*-infected macrophages can be overwhelmed by high bacterial loads, which reduces their bactericidal capacity [127, 128]. Whereas specific *S. aureus* factors were not addressed in this study, these processes are known to represent suitable targets for bacteria to promote their survival [27, 28, 129, 130]. Rodrigues-Lopes et al*.* described a similar staphylococcal phenotype deficient in the cysteine protease staphopain A [9], a protein that was expressed at steady state by all four isolates tested in this study. In mice, TNF-R1 or TNF-R2 deficiency does not alter bacterial burden but does differentially shape neutrophil defense [131]. TNF-R2 is mainly expressed on myeloid cells, but its expression has been shown to be upregulated in several tumor cell lines, including A549 [132], although this receptor and how it might indirectly shape cell death signaling [133, 134] was not investigated in the current study.

Taken together, our findings suggest that the TNF–TNF-R1 axis is a relevant determinant of U937 infection outcomes across different *S. aureus* isolates. However, the underlying mechanistic framework is likely shaped by multiple overlapping processes that remain unresolved in the current study. Pre-stimulation with TNF and the employment of different TNF antagonists will provide additional insights into protective versus adverse pathway modulation. Considering the pronounced cell-type dependence, the use of additional professional phagocytes and primary cells will be important to determine whether and how broadly these findings extend across infection-relevant tissues and whether nucleus-associated *S. aureus* interacts functionally with TNF signaling. Finally, defining the contributions of virulence factors such as protein A, SSL10 and other toxins known to mediate host cell death induction [9, 21, 71, 135], will illuminate further mechanisms beyond host determinants alone.

### Intracellular uptake and nucleo-proximal bacterial positioning

In line with previous studies [6, 9, 136], we confirmed that all analyzed isolates were rapidly internalized and primarily resided in membrane-enclosed compartments across cell lines, in which they showed signs of replication and occasionally degradation at 2 hpi. These compartments frequently demonstrated LAMP-1 decoration and commonly clustered perinuclearly, consistent with previously described spatial organization of endolysosomes to promote membrane fusion and autophagic flux [137]. However, many pathogens, including *S. aureus*, can interfere with lysosomal trafficking and fusion, as e.g. perinuclear lysosome accumulation in *S. aureus*-infected macrophages has been linked to the small GTPase Arl8 [138].

Our findings indicate common association of bacteria, primarily within membrane-bound vacuoles, with host nuclear structures in U937, A549, and SaOS-2 cell lines. Peri- and juxtanuclear localization becomes apparent (although not acknowledged as such) in other TEM studies of *S. aureus*-infected host cells, demonstrating bacteria-containing invaginations of the nuclear membrane or bacteria-containing vacuoles associated with the nuclear structures also at lower MOIs than those of present study [139, 140].

Interestingly, our transcriptomic analysis identified ‘Nuclear localization signal (NLS)-bearing nuclear import’ as a major downregulated pathway across all isolates in U937 monocytes. Whether nucleo-proximal positioning affects nuclear processes or involves translocation of bacterial proteins such as nucleomodulins [141–145] was not determined in the current study.

Generally, nuclear access has been described for protozoa, while few reports have shown nuclear localization or close contact of bacteria with the nuclear membrane in mammalian cells*,* e.g. for *Mycoplasma genitalium* and *Burkholderia pseudomallei,* or the human pathogens *L. monocytogenes*, *O. tsutsugamushi* or *R. rickettsii* [146–151]*.* Chamberlain and colleagues reported similar findings of bacterial association with nuclear structures in human phagocytic (U937, THP-1) and non-phagocytic (HeLa) cell lines and argued the possibility of nuclear access for the amoeba-associated intranuclear bacterium ‘*Candidatus Berkiella cookevillensis’*, however at lower rates compared to their amoeba host [152]. The authors suggest that nuclear positioning could be based on deep invaginations of the nuclear membranes, which closely resemble the nuclear invaginations associated with *S. aureus*-containing vesicles observed in our study, thereby surrounding itself with the host membrane. Supposedly, these structures may also reflect a cellular stress response comparable to that observed during heat stress, in which deep invaginations have been described as a distinct functional compartment associated with intermediate filament collapse, organelle clustering, concentrated de novo protein synthesis, and increased protein degradation [153]. Whereas SBF-SEM findings supported nucleo-proximal localization across several sections, the surrounding electron-translucent compartment observed in some TEM sections could not be resolved. A similar state was occasionally observed also for *Cand. B. cookevillensis* [152].

Importantly, while our analyses clearly suggest relevant association of bacteria-containing and nuclear compartments, there are several major limitations. First, imaging did not show nuclear envelope integrity required to allow for accurate positioning of the observed structures. Lamin staining attempts during *S. aureus* infection were limited due to unspecific labeling of the bacterial surface, possibly due to *S. aureus* protein A [154–156] that is upregulated in an agr-controlled manner during early infection phases [157], and thus requires careful optimization of protocols. Second, whereas we could also identify viable *S. aureus* in the nuclear fraction of each tested cell line, all nuclear fractionation protocols require centrifugation steps, which entail the risk of bacterial co-sedimentation. Hence, these observations demand careful analysis of the morphological and functional organelle integrity of the derived fractions, and may benefit from the use of (immune-) magnetic labeling and isolation strategies, such as lipobiotin labeling as introduced above [43, 158]. Finally, additional spatiotemporal analyses, including nuclear envelope as well as inner membrane integrity and distortion, the composition of bacteria-containing compartments, and their possible interaction at different stages of infection are essential to determine whether this noncanonical nucleo-proximal localization represents an occasional, transient trafficking stage or a distinct niche supporting persistence, growth, or host modulation.

## Conclusions

Together, our findings show that intracellular *S. aureus* infection is shaped by the interaction between bacterial isolate, host-cell type, and TNF-R1–associated regulation of U937 cell fate. The isolates induced a shared early transcriptional stress response but differed substantially in their broader host transcriptional programs and cell-death–associated responses. In U937 monocytes, TNF-R1 influenced the timing and magnitude of these responses, whereas its contribution was less pronounced in A549 and SaOS-2 cells. This work warrants further investigation of TNF-R1–mediated antimicrobial functions that might translate to infection risks under TNF/TNF-R1–targeted therapies. Across the investigated cell types, bacteria were frequently observed in close association with nuclear structures, the functional relevance of which remain to be established.

## Supplementary Information

Supplementary Material 1.

## Data Availability

Data and materials availability: All data needed to evaluate the conclusions in this paper are present either in the main text, in the supplementary materials.

## Abbreviations

ΔΨm: Mitochondrial transmembrane potential
7-AAD: 7-Amino-actinomycin D
ADAM17/TACE: A disintegrin and metalloproteinase 17/Tumor necrosis factor-α converting enzyme
agr: Accessory gene regulator
AWB: Apoptosis Wash Buffer
bc: Boyden chamber
CFU: Colony-forming units
CHX: Cycloheximide
COX1: Cytochrome C oxidase 1
DEG: Differentially expressed gene
DPEP2: Dipeptidase 2
FASP: Filter-aided sample preparation
GO:BP: Gene ontology biological processes
hpi: Hours post infection
LAMP: Lysosome-associated membrane protein
LB: Luria-Bertani broth
Log2FC: Log2-transformed fold change
MAPK: Mitogen-activated protein kinases
Min_adj_score: Minimal adjusted enrichment score
MOI: Multiplicity of infection
nCI: Normalized cell index
NF-κB: Nuclear factor κB
OD_600_: Optical density at 600 nm
Padj: Adjusted p-value
PARP-1: Poly(ADP)ribose-polymerase 1
PCA: Principal component analysis
PI: Propidium iodide
PTMs: Post-translational modifications
*S.**aureus*: *Staphylococcus* *aureus*
SBF-SEM: Serial block face scanning electron Microscopy
SIM: Structured illumination microscopy
SSL-10: Staphylococcal superantigen-like protein 10
TEM: Transmission electron microscopy
TNF: Tumor necrosis factor α
TNF-R1/-R2: TNF-receptor 1/-2
zVAD.fmk: Benzyloxycarbonyl-Val-Ala-Asp(OMe)-fluoromethylketone [Pan-caspase inhibitor]
