# Supplementary material for: Intracellular Staphylococcus aureus triggers isolate-specific host transcriptional responses alongside TNF-R1 regulated cell fate decisions
Source: Cell Commun Signal. 2026 Sep 28;24:515. doi: 10.1186/s12964-026-03246-8 (PMC13621601; doi:10.1186/s12964-026-03246-8)

Uncropped WB‘s Fig. 1K

A549wt, 2hpi

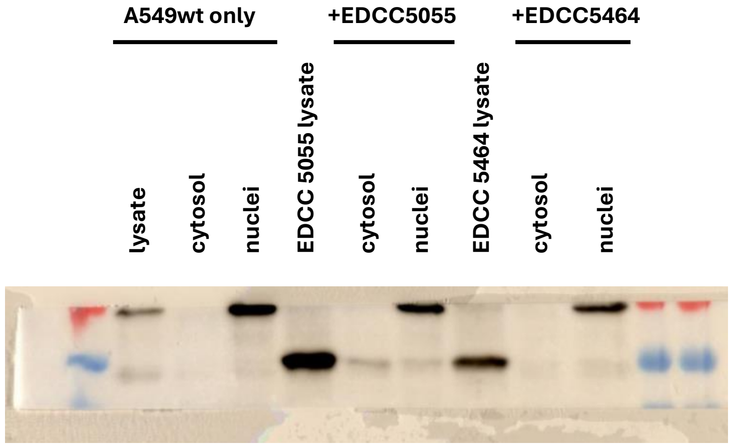

Lamin B1

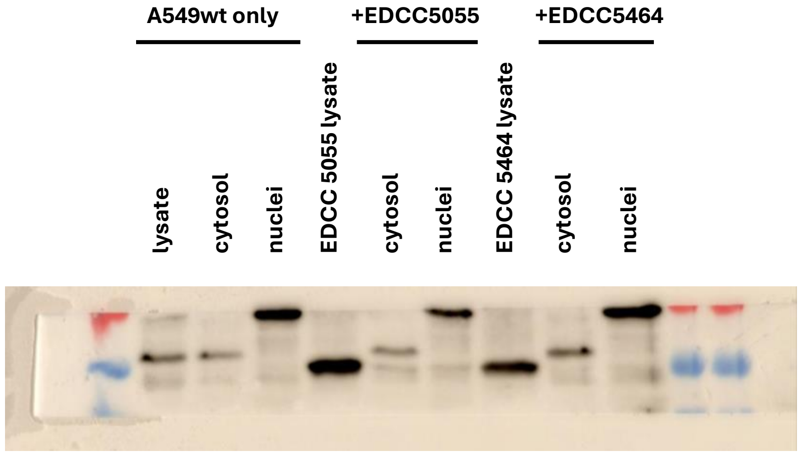

α-tubulin

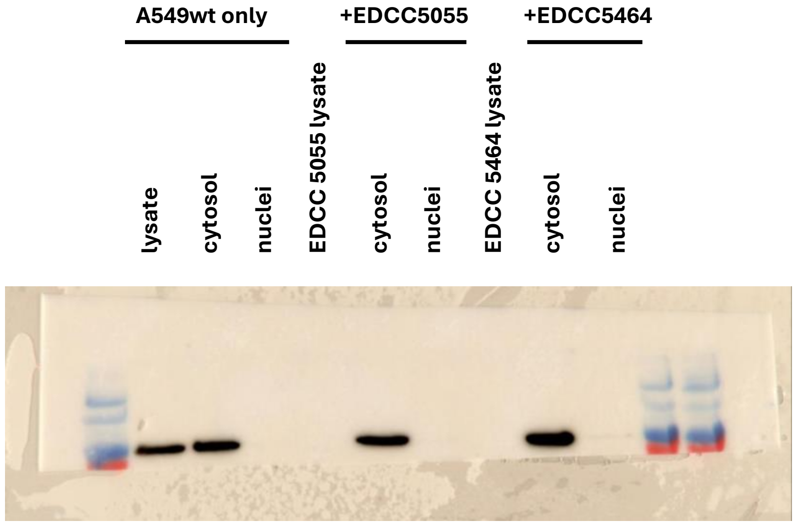

HSP90β

A549wt, 2hpi

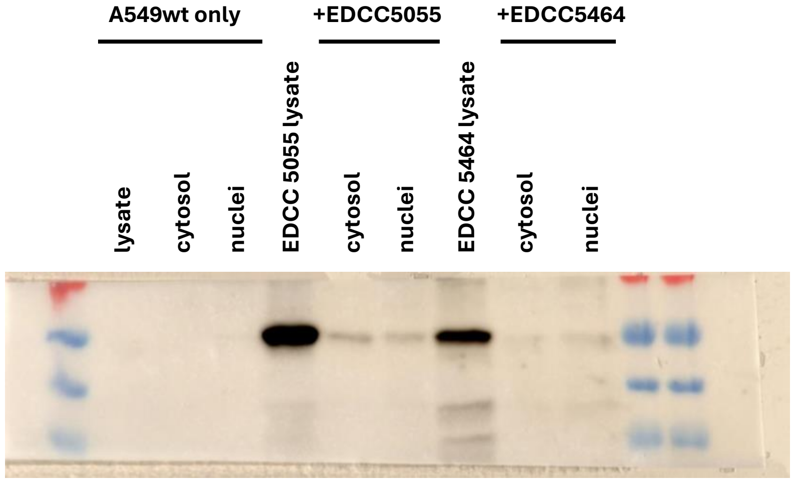

S. aureus **(pan)**

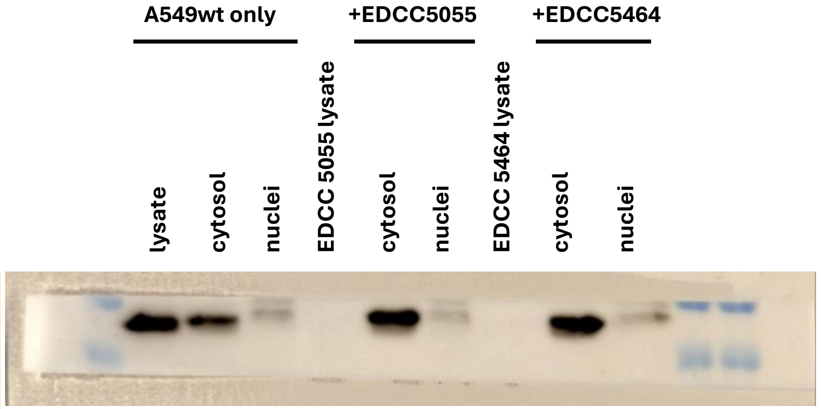

GAPDH

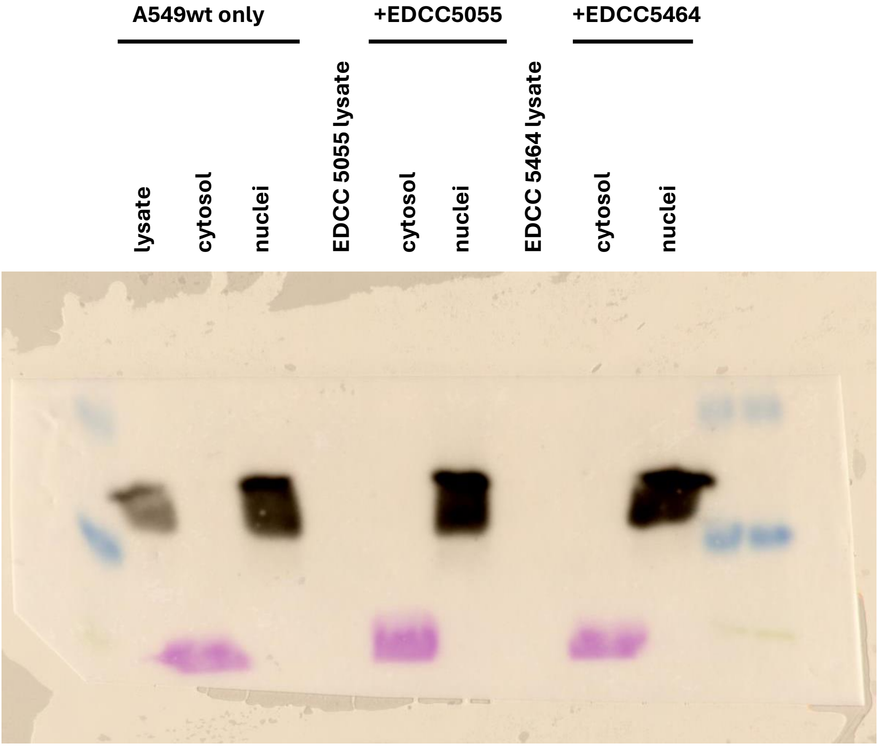

Histone H3

U937wt, 2hpi

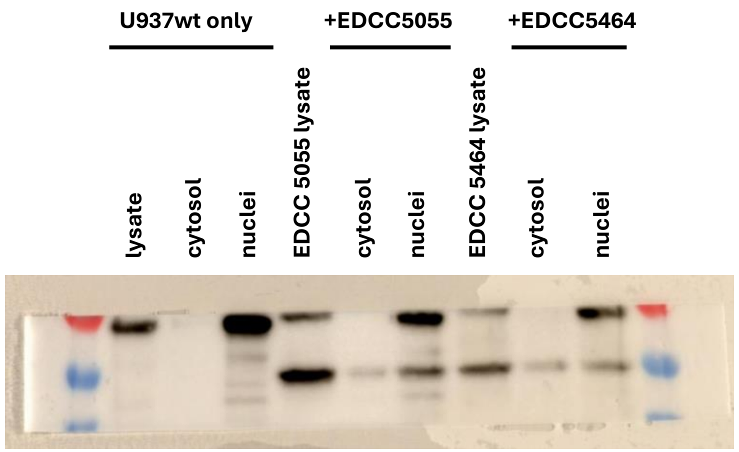

Lamin B1

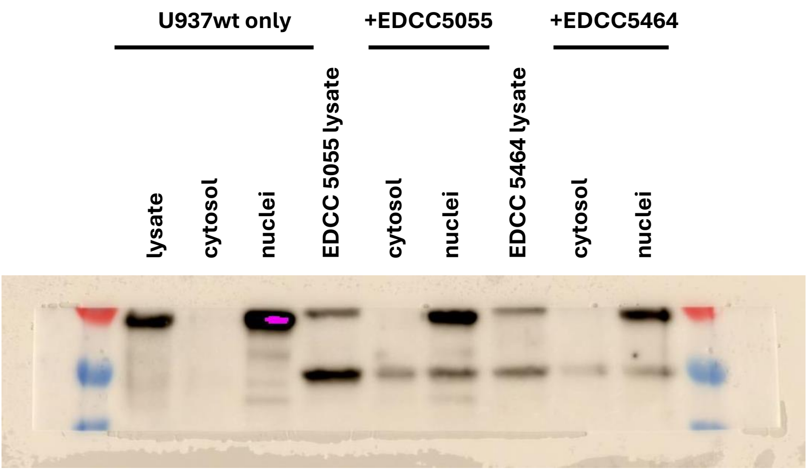

α-tubulin

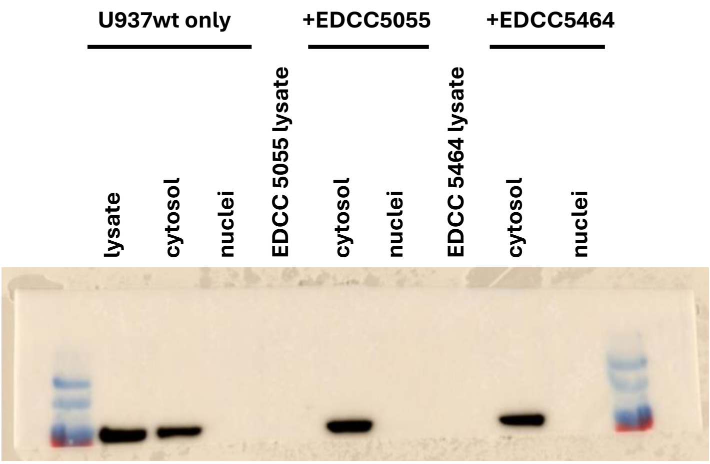

HSP90β

U937wt, 2hpi

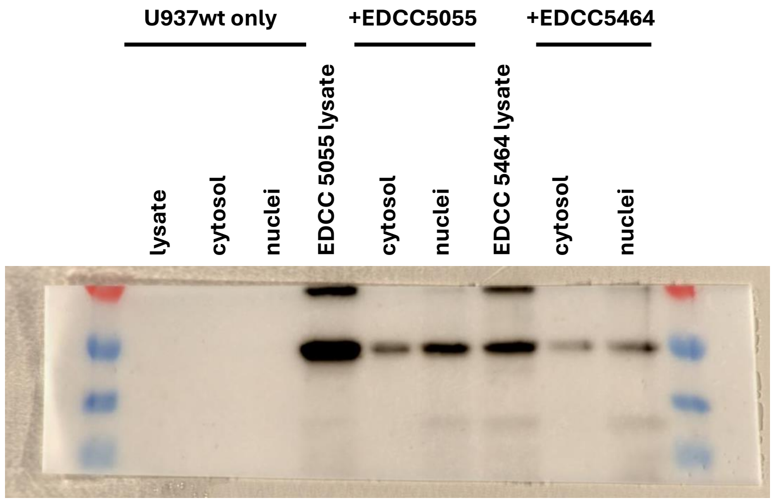

S. aureus **(pan)**

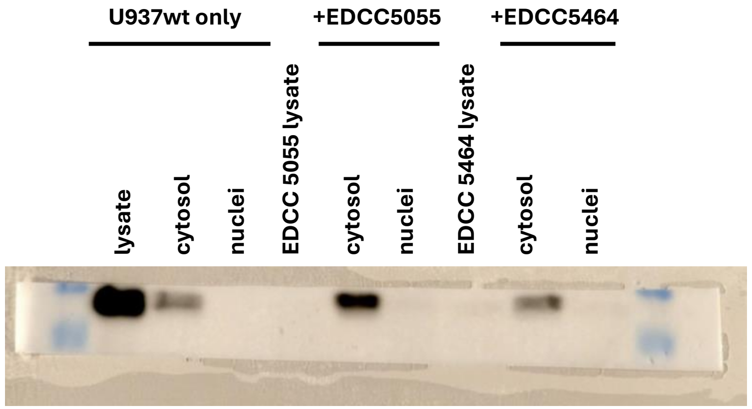

GAPDH

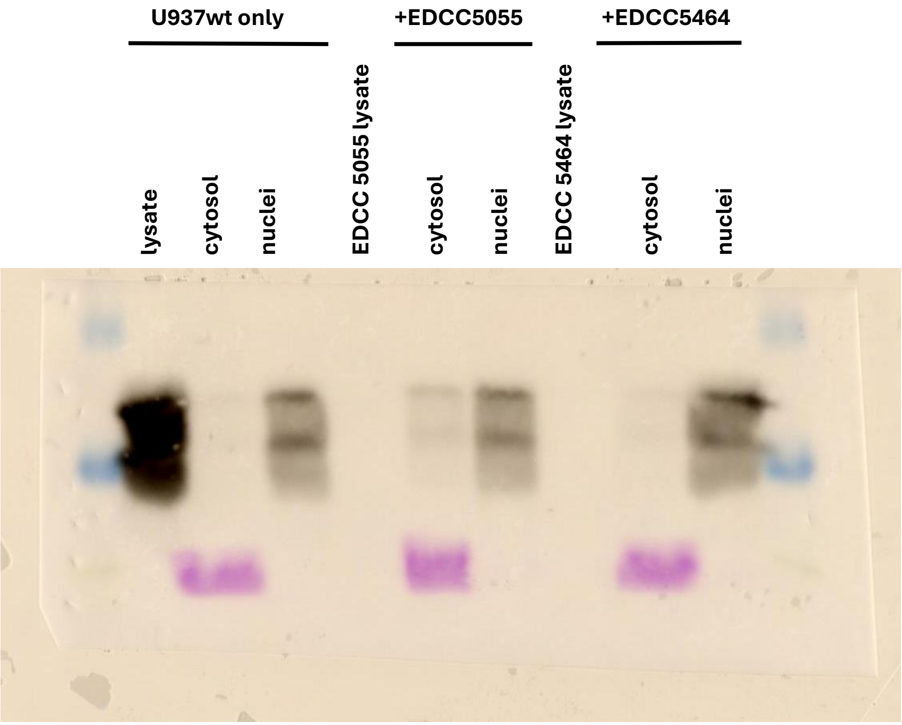

Histone H3

SAOS-2wt, 2hpi

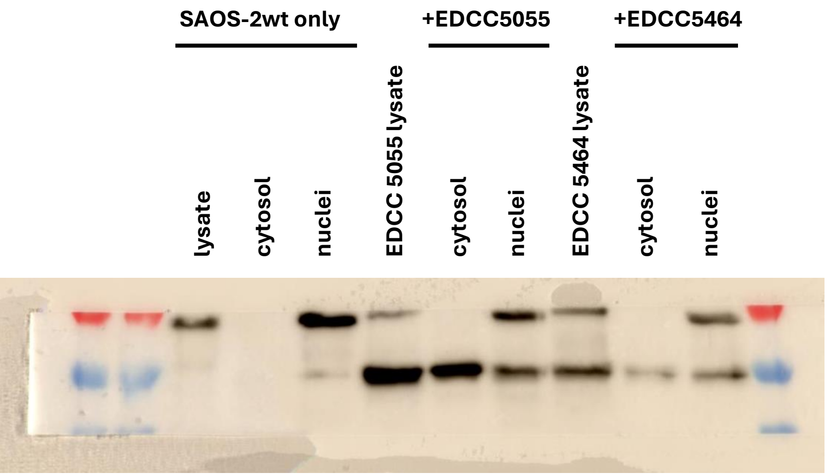

Lamin B1

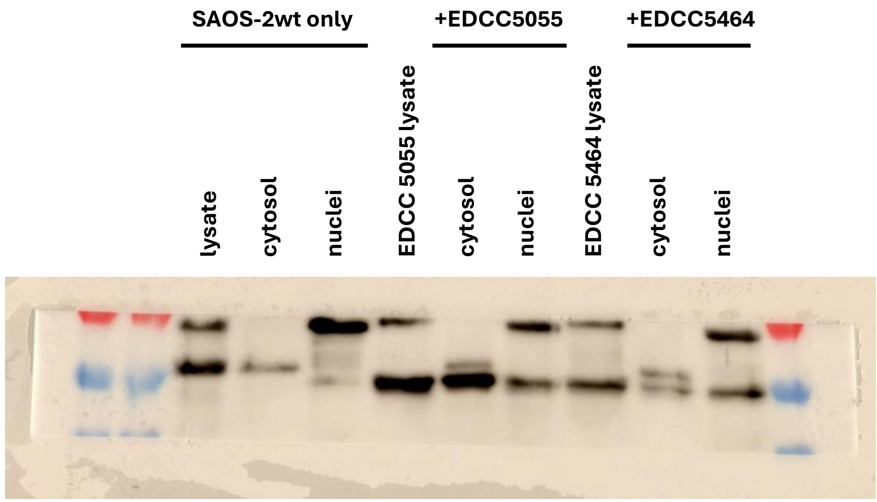

α-tubulin

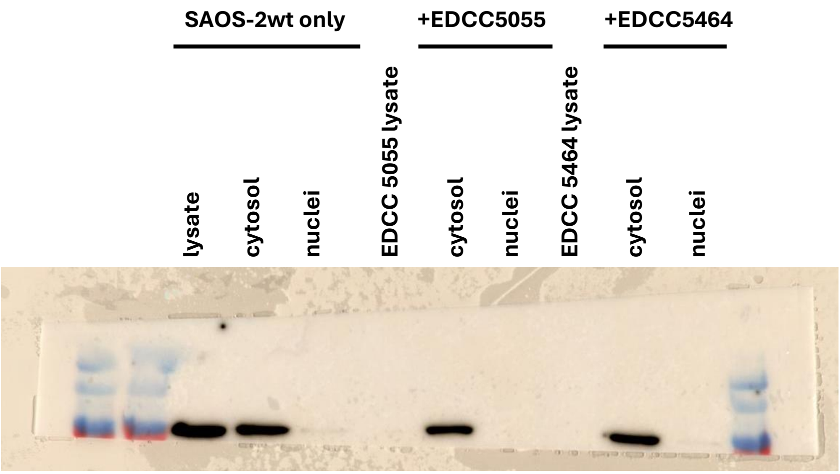

HSP90β

SAOS-2wt, 2hpi

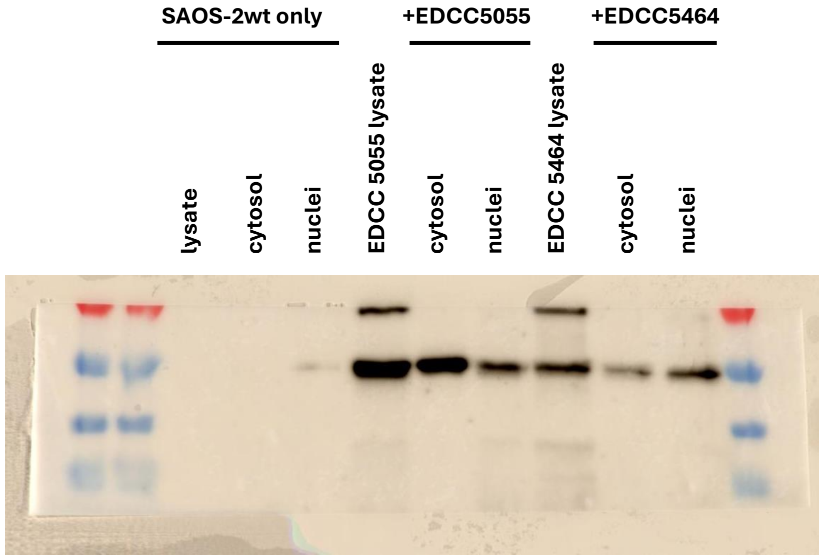

S. aureus **(pan)**

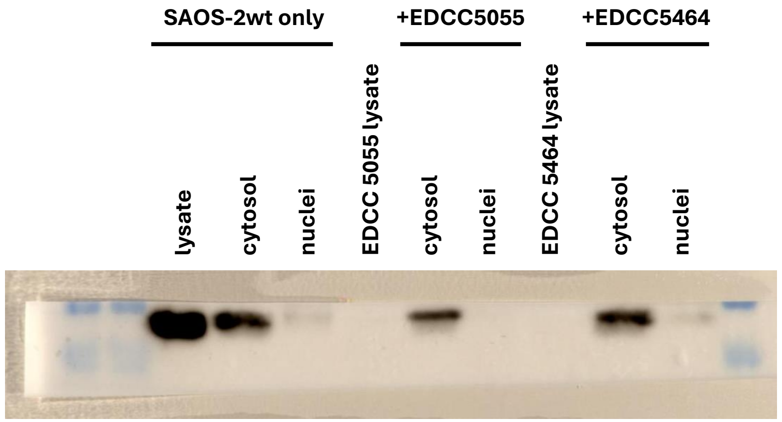

GAPDH

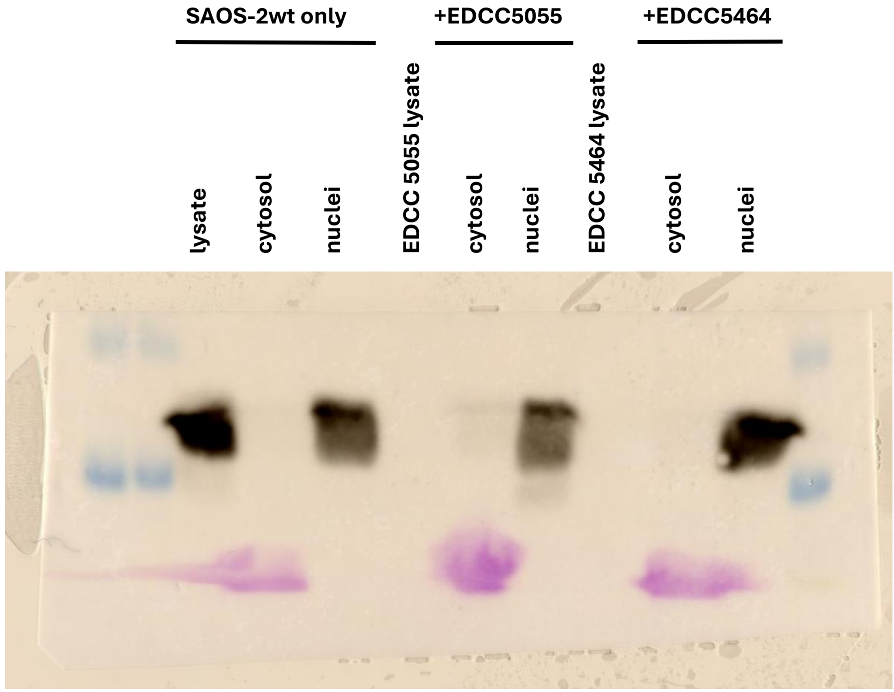

Histone H3

Uncropped WB‘s Fig. 4E + F

U937 wt, 6hpi

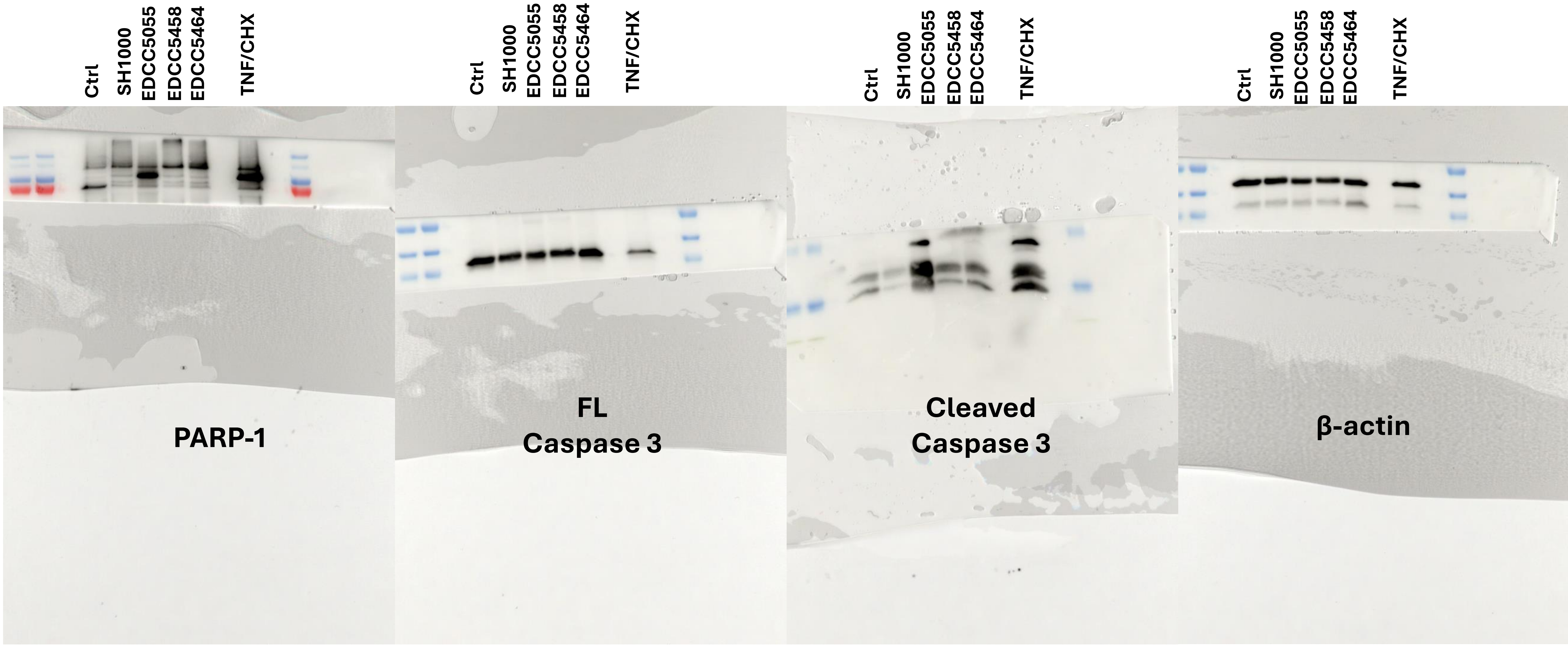
Ctrl
SH1000
EDCC5055
EDCC5458
EDCC5464
TNF/CHX
PARP-1
FL
Caspase 3
Cleaved
Caspase 3
β-actin

U937 wt, 24hpi

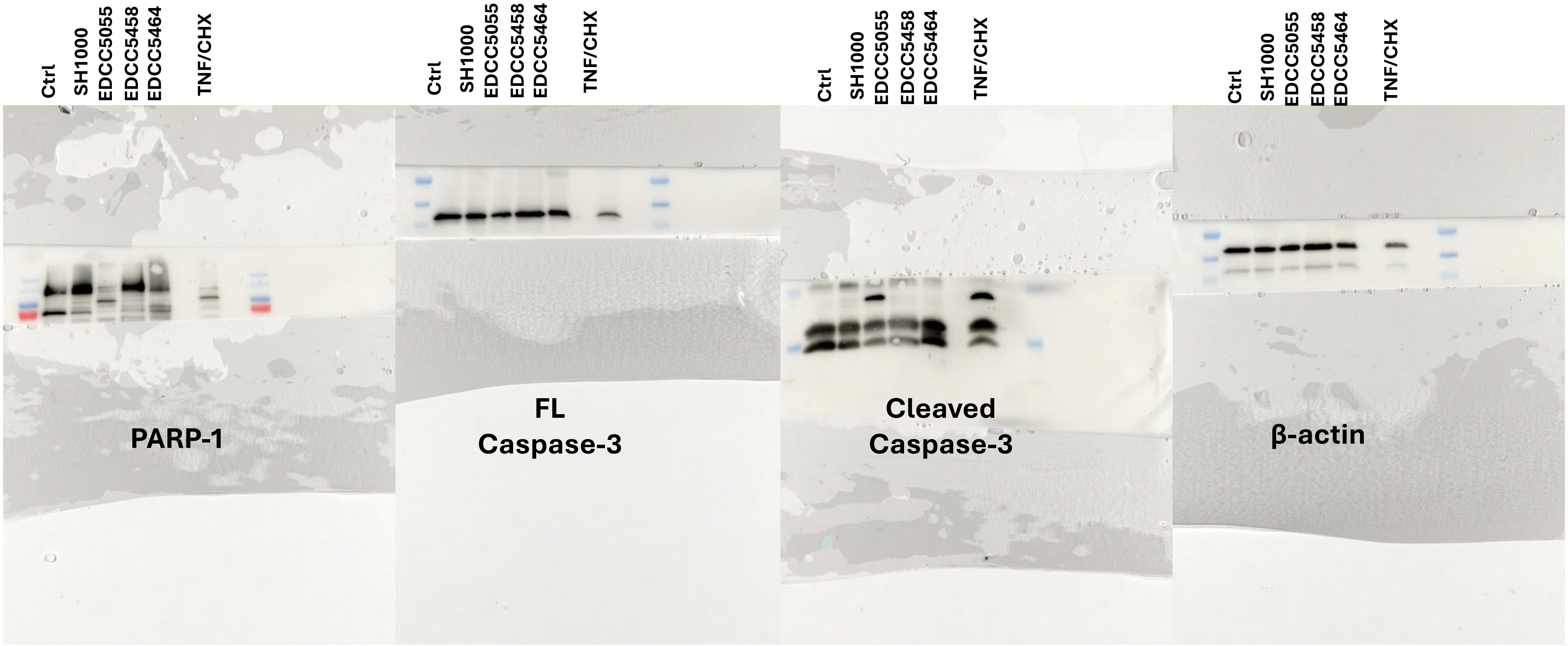

Supplement: Supplementary file 1 [file 12964_2026_3246_MOESM1_ESM.zip › 20260702 Western Blot Raw Images.pdf]
