# Supplementary material for: Intracellular Staphylococcus aureus triggers isolate-specific host transcriptional responses alongside TNF-R1 regulated cell fate decisions
Source: Cell Commun Signal. 2026 Sep 28;24:515. doi: 10.1186/s12964-026-03246-8 (PMC13621601; doi:10.1186/s12964-026-03246-8)
Supplement: Supplementary file 1 [file 12964_2026_3246_MOESM1_ESM.zip › 20260702 all Supplements.zip!/Supplements/S6_DESeq2_comparison_EDCC5458_4h_vs_untreated_interactive_plots.html]

EDCC5458\_4h vs untreated
