# Supplementary material for: Intracellular Staphylococcus aureus triggers isolate-specific host transcriptional responses alongside TNF-R1 regulated cell fate decisions
Source: Cell Commun Signal. 2026 Sep 28;24:515. doi: 10.1186/s12964-026-03246-8 (PMC13621601; doi:10.1186/s12964-026-03246-8)
Supplement: Supplementary file 1 [file 12964_2026_3246_MOESM1_ESM.zip › 20260702 all Supplements.zip!/Supplements/supplementary figures_revised/Figure S10_revised.pdf]

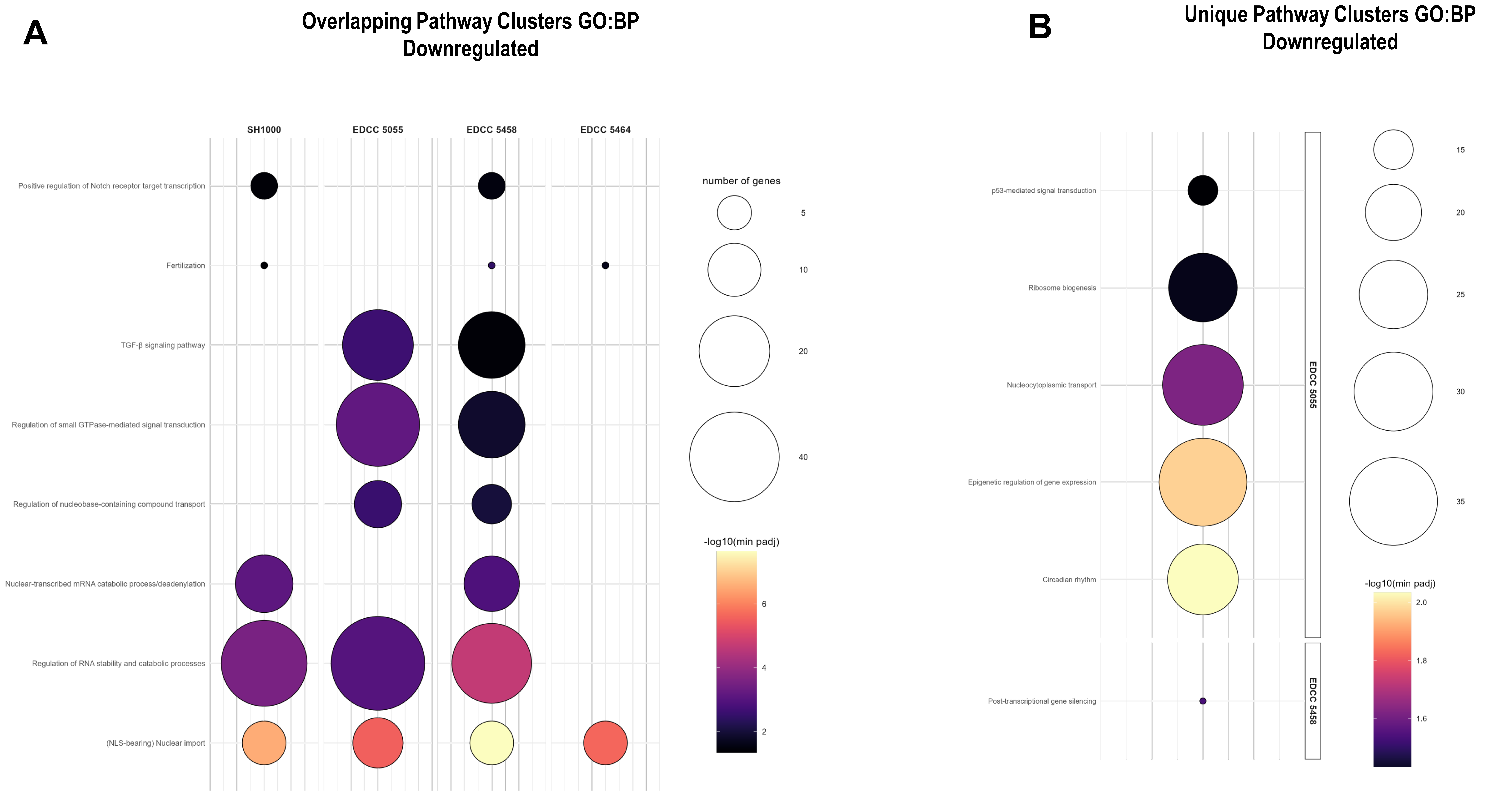

Fig. S10 Intracellular *S. aureus* infection of U937 cells induces distinct downregulated pathway clusters dependent on each isolate. Significantly (padj≤0.05), at least two-fold upregulated protein-coding genes were analyzed for enriched pathways (Gene Ontology Biological Processes) using g:Profiler ordered query, and clustered based on Jaccard similarity of gene sets, using hierarchical clustering (average linkage) and a fixed cut height of 0.5. The adjusted enrichment score of a driver pathway (minimal padj) is shown representatively for each cluster (-log10(min_adj_score)) and bubble size indicates gene set size, for (**A**) overlapping and (**B**) unique pathway clusters of *S. aureus* isolates compared to untreated controls.
