# Supplementary material for: Intracellular Staphylococcus aureus triggers isolate-specific host transcriptional responses alongside TNF-R1 regulated cell fate decisions
Source: Cell Commun Signal. 2026 Sep 28;24:515. doi: 10.1186/s12964-026-03246-8 (PMC13621601; doi:10.1186/s12964-026-03246-8)
Supplement: Supplementary file 1 [file 12964_2026_3246_MOESM1_ESM.zip › 20260702 all Supplements.zip!/Supplements/supplementary figures_revised/Figure S12_revised.pdf]

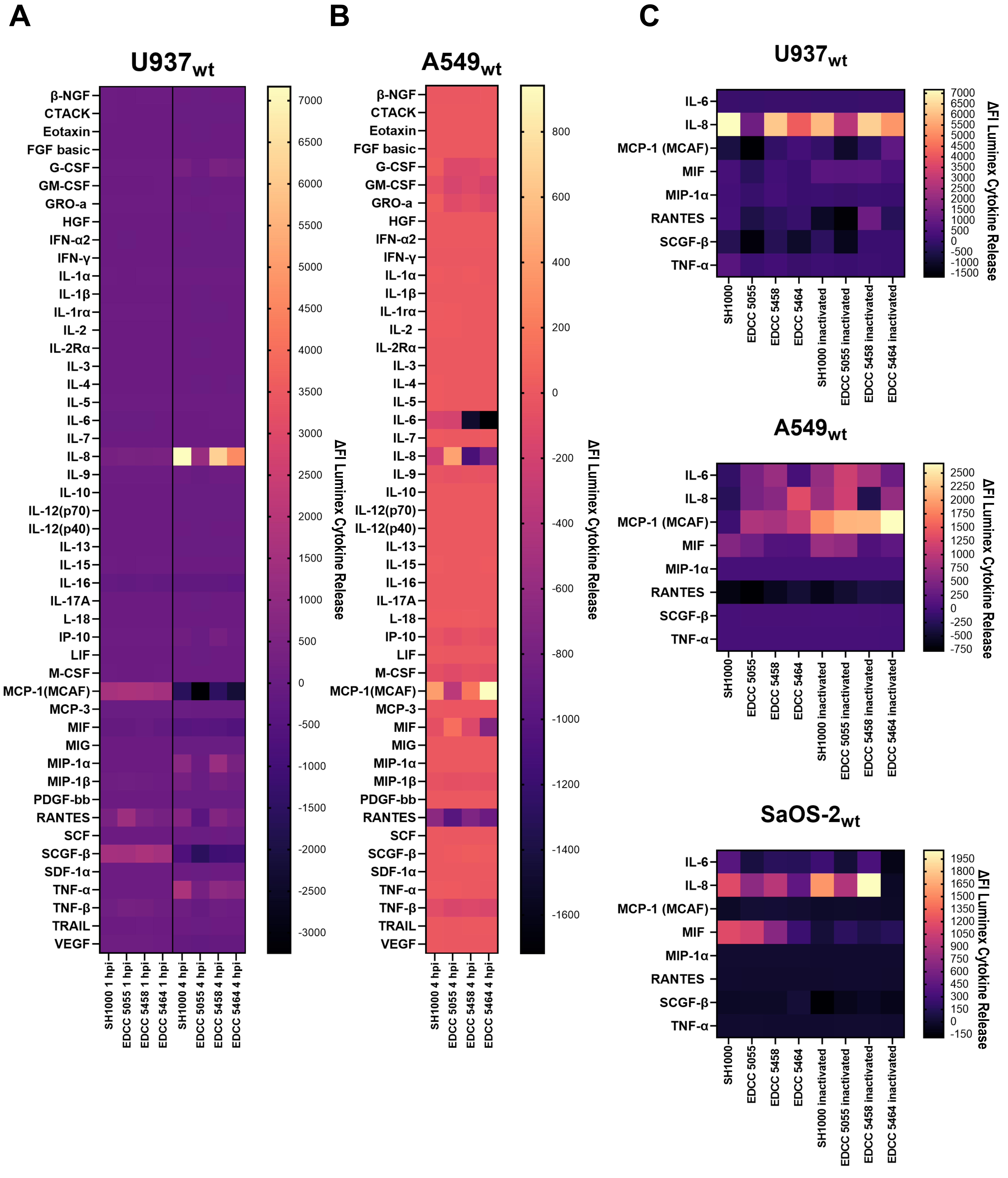

Fig. S12 Luminex ELISA of *S. aureus*-infected (**A**) U937wt at 1 hpi and 4 hpi and (**B**) A549wt cells at 4 hpi (n=1), MOI 30. (**C**) Median fluorescence intensities of eight selected cytokines using Luminex for U937, A549 (n=3 for viable, n=2 for inactivated S. aureus), and SaOS-2 (n=1) cells**.** Fluorescence intensities normalized to uninfected control cells and background (**ΔFI**).
