# Supplementary material for: Intracellular Staphylococcus aureus triggers isolate-specific host transcriptional responses alongside TNF-R1 regulated cell fate decisions
Source: Cell Commun Signal. 2026 Sep 28;24:515. doi: 10.1186/s12964-026-03246-8 (PMC13621601; doi:10.1186/s12964-026-03246-8)
Supplement: Supplementary file 1 [file 12964_2026_3246_MOESM1_ESM.zip › 20260702 all Supplements.zip!/Supplements/supplementary figures_revised/Figure S13_revised.pdf]

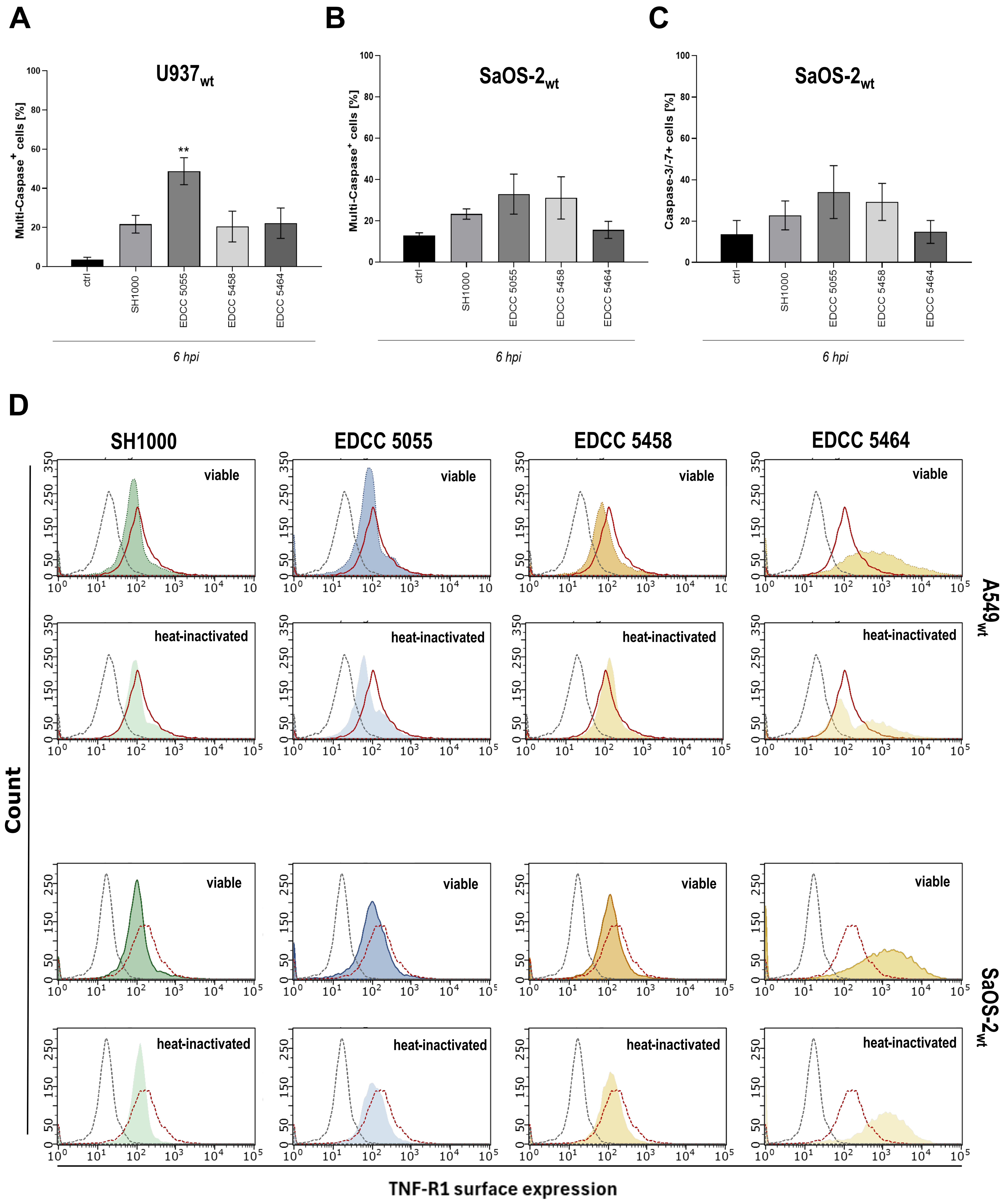

Fig. S13 Flow cytometry analysis of activity of multiple caspases in (**A**) U937wt and (**B**) SaOS-2 cells and (**C**) caspase-3/-7 in SaOS-2 cells upon *S. aureus* infection at 6 hpi following a gentamicin protection assay. Data show mean ± SEM of n≥3 independent experiments. (**D**) TNF-R1 surface expression in uninfected (grey: unlabelled; red: labelled) and infected A549 (upper panel) and SaOS-2 (lower panel) cells with viable or heat-inactivated *S. aureus* at 2 hpi. Statistical analysis was performed using One-way ANOVA with Tukey's Multiple Comparisons comparing means between all conditions. Signifance is shown for comparison against uninfected controls. **p<0.01.
