# Supplementary material for: Intracellular Staphylococcus aureus triggers isolate-specific host transcriptional responses alongside TNF-R1 regulated cell fate decisions
Source: Cell Commun Signal. 2026 Sep 28;24:515. doi: 10.1186/s12964-026-03246-8 (PMC13621601; doi:10.1186/s12964-026-03246-8)
Supplement: Supplementary file 1 [file 12964_2026_3246_MOESM1_ESM.zip › 20260702 all Supplements.zip!/Supplements/supplementary figures_revised/Figure S3_revised.pdf]

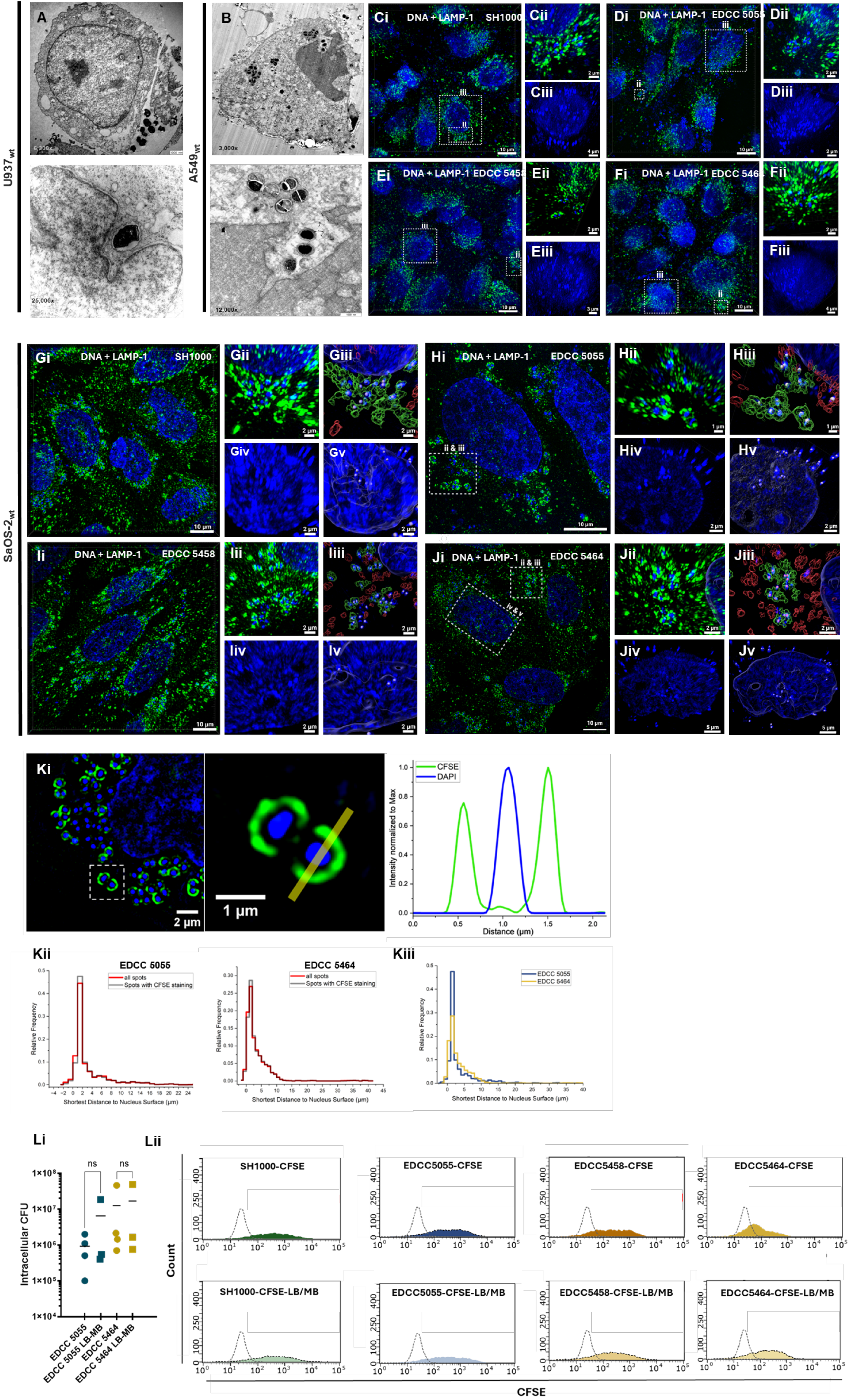

Fig. S3 Peri- and putative intranuclear, LAMP-1 associated localization of *S. aureus* at 2 hpi and MOI 100. Each cell line was infected with *S. aureus* at MOI 100 using a gentamicin protection assay and cells were fixed at 2 hpi. Transmission electron microscopy of peri- and intranuclear localization (**A**) EDCC 5055 in U937 and (**B**) EDCC 5464 in A549 cells. 3D-rendering of (**C-F**) A549 and Maximum intensity projection of (**G-J**) SaOS-2 cells infected with *S. aureus* (**C, G**) SH1000, (**D**) EDCC 5055, (**E, H**) EDCC 5458, and (**F**) EDCC 5464 imaged by SIM. DNA was stained by DAPI (blue) and LAMP-1 was stained by mouse anti-LAMP1 and AF488 (green). (**Cii/iii – Jii/iii**) Zoom-in of boxed region in Ci-Fi. (**Gii/iii, JHii/iii**) Surfaces and spots generated after image analysis in Imaris (11.0.1). Surfaces defining the nucleus are visualized by a blue outline, spots corresponding to bacterial DNA are highlighted by white spheres. Surfaces generated around LAMP1 signal are classified based on distance to the closest bacterial spot. Surfaces closer than 0.25 µm coloured in green, while surfaces at a greater distance are coloured in red. (**Giv- Jiv**) DAPI stained nucleus of a cell infected with EDCC 5055/5464 (not depicted in Gi). Strong DAPI staining consistent with bacterial DNA stain can be observed inside the nucleus or located within an invagination of the nuclear lamina. (**Gv- Jv**) Spots are identified inside the generated nucleus surface. (**Ki**) Bacterial DNA spot size selection based on CFSE-stained (green) *S. aureus* to exclude misclassification due to heterochromatin-dense nuclear spots, showing (**Kii**) reproducible bacterial spot identification. (**Kiii**) Histogram of shortest distances between identified CFSE-labelled bacterial spots and the surface generated around the nucleus (bin size 1 µm; 8 images per strain; 1515 ± 160 (mean ± SD) detected spots per strain; images see Fig. 1).(**Li**) Intracellular CFU Count and (**Lii**) Flow Cytometry Analysis of U937 cells infected with unlabelled or lipobiotin/streptavidin-magnetic beads-labelled *S. aureus* (LB-MB) used for TEM imaging.
